# Supplementary material for: Apis mellifera Solinvivirus-1, a Novel Honey Bee Virus That Remained Undetected for over a Decade, Is Widespread in the USA
Source: Viruses. 2023 Jul 21;15(7):1597. doi: 10.3390/v15071597 (PMC10384192; doi:10.3390/v15071597)

**Supplementary Figure S2**. Binomial modeling results AmSV1 prevalence across US climate zones (based on https://www.researchgate.net/figure/US-climate-regionsidentified-by-the-National-Climate-Data-Center-adapted-from-Karl-and_fig1_306023638 )

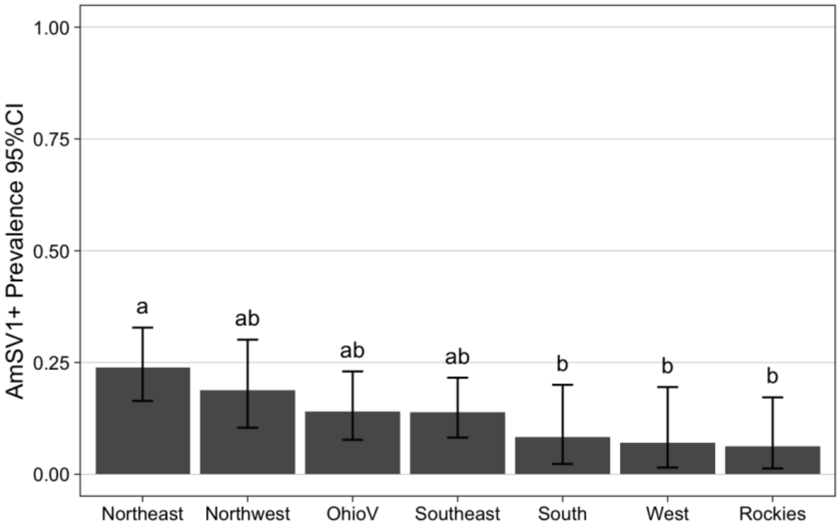

Supplement: Supplementary file 1 [file viruses-15-01597-s001.zip › Supplementary-Figure S2-AmSV1 in US regions.pdf]
