# Supplementary material for: Apis mellifera Solinvivirus-1, a Novel Honey Bee Virus That Remained Undetected for over a Decade, Is Widespread in the USA
Source: Viruses. 2023 Jul 21;15(7):1597. doi: 10.3390/v15071597 (PMC10384192; doi:10.3390/v15071597)
Supplement: Supplementary file 1 [file viruses-15-01597-s001.zip › Supplementary-Figure-S1-Protein-Alignment-Solinviviruses.pdf]

Supplementary Figure S1. Alignment of the complete polyprotein sequences of *Apis mellifera* solinvivirus-1 (AmSV1) and classified and putative solinviviruses. GenBank accession numbers are shown, for Rosy apple aphid virus and Solenopsis invicta virus-3, the putative ORF1-ORF2 fusion products were used.

```
DQ286292 Rosy apple aphid virus                       ------------------------------------------------------------ 0
OP265432 Penaeus vannamei solinvivirus                -----------------------MQSEEL------------------------------- 6
MZ210029 Fushun monoleptalauta Solinvi-like virus 1   ------------------------------------------------------------ 0
KX024775 Nylanderia fulva virus 1                     -----------------------MTSEKVMRIRGERA-------------GTKPIGDVME 24
KY070327 Diabrotica virgifera virgifera virus 2       -----MFNPKCLT--PKVNITSVMQTPKVATPKVATPKVTPKVNEIDPTIN-KITAILMI 52
OQ540582 Apis mellifera solinvivirus-1                MSYIDMNAEKQTNAEKHTNAERHMNAEKQQNA----EKHTNAEKHMNAEKHKQTKNAEMH 56
MN918666 Picornavirales [unknown host].               ------------------------------------------------------------ 0
MW251313 Icha Creek insect virus                      ------------------------------------------------------------ 0
MZ822083 Apis picorna-like virus-4                    ------------------------------------------------------------ 0
FJ528584 Solenopsis invicta virus-3                   ------------------------------------------------------------ 0
MW314636 Myrmica rubra picorna-like virus             ------------------------------------------------------------ 0
OL569508 Corparats virus-2                            MPYVRDLEDYE-------------------------------FWEVEPE----PISFEVE 25


DQ286292 Rosy apple aphid virus                       ------------------------------------------------------------ 0
OP265432 Penaeus vannamei solinvivirus                --------YKLSHCKVKSCISFALYKRHSC------------------------------ 28
MZ210029 Fushun monoleptalauta Solinvi-like virus 1   ---------------MRDCLKRFY-STFIGIYSALVGTSKQQNSTSNGIENSTSIGNYNS 44
KX024775 Nylanderia fulva virus 1                     LNS-----FETI-----------------F------------------------------ 32
KY070327 Diabrotica virgifera virgifera virus 2       MQIGTILLFKTTRNSIFSCLQRTY-SKFSL-Y---------------------------- 82
OQ540582 Apis mellifera solinvivirus-1                MKAGKHCVSKHILSRARVLQARKY-KRKEL-NLPWNE----------------------- 91
MN918666 Picornavirales [unknown host]                ------------------------------------------------------------ 0
MW251313 Icha Creek insect virus                      ------------------------------------------------------------ 0
MZ822083 Apis picorna-like virus-4                    ------------------------------------------------------------ 0
FJ528584 Solenopsis invicta virus-3                   ------------------------------------------------------------ 0
MW314636 Myrmica rubra picorna-like virus             ------------------------------------------------------------ 0
OL569508 Corparats virus-2                            VVHNTMPYFKHLQHTVKEWKGWTR-EEASFFISEWID----------------------- 61


DQ286292 Rosy apple aphid virus                       ------------------------------------------------------------ 0
OP265432 Penaeus vannamei solinvivirus                ----TRMTPHPSNLKPLPDLDALA-------DANIQEATLEY-----FEEDFLCLQHIPS 72
MZ210029 Fushun monoleptalauta Solinvi-like virus 1   TSNGTFVHRKPKHIFNGSGIHTRLMMYTFIMTTMAMKRTMKYRQIPSSIGLFDHRITPIS 104
KX024775 Nylanderia fulva virus 1                     -------VMVPLVVAHVRSFVTRMFS--------------------TQFTVGGVRQ--KS 63
KY070327 Diabrotica virgifera virgifera virus 2       ----------------------------------------------SELAIFGTFITLCV 96
OQ540582 Apis mellifera solinvivirus-1                -------IR-------------------------------------DKITPDFAFILGCS 107
MN918666 Picornavirales [unknown host]                ------------------------------------------------------------ 0
MW251313 Icha Creek insect virus                      ---------------------MCLMI--------------------PQLTFVFCFV---- 15
MZ822083 Apis picorna-like virus-4                    ------------------------------------------------------------ 0
FJ528584 Solenopsis invicta virus-3                   ------------------------------------------------------------ 0
MW314636 Myrmica rubra picorna-like virus             ------------------------------------------------------------ 0
OL569508 Corparats virus-2                            -------HPSPFNPKNYADFVEKSWM--------------------AQFDDFFAFV---- 90


DQ286292 Rosy apple aphid virus                       ------------------------------------------------------------ 0
OP265432 Penaeus vannamei solinvivirus                SSDLEFLADNES----------------------------------ARALCESMDEMQEH 98
MZ210029 Fushun monoleptalauta Solinvi-like virus 1   TIALQMYNIRWKTLPPWKPFKKIRKLR--------------------------------K 132
KX024775 Nylanderia fulva virus 1                     SRTLAFFQKFRTGS---VRVRKYMRTNVEMVEKVVEPQNSEPVELD------GVN----- 109
KY070327 Diabrotica virgifera virgifera virus 2       LPSVGFLSLIITAP---IIVKKIKKTK-IQKHPLHNF------------TC--------- 131
OQ540582 Apis mellifera solinvivirus-1                I-----YPFLYPAS---KLFERFDKRR-QMIKNFNNLQ---------NEAN--------- 140
MN918666 Picornavirales [unknown host]                ------------------------------------------------------------ 0
MW251313 Icha Creek insect virus                      -I---FAALL---P------------EYRYKIALKNRKR-EVFDDSGKDPGKGLFQ---D 52
MZ822083 Apis picorna-like virus-4                    ------------------------------------------------------------ 0
FJ528584 Solenopsis invicta virus-3                   ------------------------------------------------------------ 0
MW314636 Myrmica rubra picorna-like virus             ------------------------------------------------------------ 0
OL569508 Corparats virus-2                            -SNLGFQNLK---P---IHFKKEKPQPYSIAIPVHENRFSILPESSGGDVNNGWKA---S 140


DQ286292 Rosy apple aphid virus                       ------------------------------------------------------------ 0
OP265432 Penaeus vannamei solinvivirus                LCHPAVEHLDHVPCFFPEQNLRPA---------TSAW--QALLLDEHIARWAHVPLRHRT 147
MZ210029 Fushun monoleptalauta Solinvi-like virus 1   QSES---LIDTLKDYFISNRVDLALSVILATSIVQMVSTIV----EMFRYKN----AKQQ 181
KX024775 Nylanderia fulva virus 1                     QANE---PMSVVDTLISAAAVLIGIPLSTACTISSMLVNISMLLWEFLT-DK----TSIK 161
KY070327 Diabrotica virgifera virgifera virus 2       EGDN---LITDLKNFLVPMLETYALPVAYIATLASSLVTVFALTKHYISTPK----KKRT 184
OQ540582 Apis mellifera solinvivirus-1                QETL---EVKSITQFIMTCLETFALPIIYITTLTSSLIGLITSIKTYLIMRK----DSPK 193
MN918666 Picornavirales [unknown host]                ------------------------------------------------------------ 0
MW251313 Icha Creek insect virus                      FPQE---MIKHFETVITPCFTVANFPVTAVANLVSASTTMLYVFGQIIRTWS----KSKE 105
MZ822083 Apis picorna-like virus-4                    ----------------MECVNSLNP-SDT------H--------------H--------- 14
FJ528584 Solenopsis invicta virus-3                   ------------------------------------------------------------ 0
MW314636 Myrmica rubra picorna-like virus             ------------------------------------------------------------ 0
OL569508 Corparats virus-2                            LSKP---K-----VVYKKKTKKLNP-RKVNYRLKSHKIKNIQLVHQGLLSW--------- 182


DQ286292 Rosy apple aphid virus                       ------------------------------------------------------------ 0
OP265432 Penaeus vannamei solinvivirus                LARWNSTFHETCAELNLPPYFTPTVQRQSISDIILDVIKSELVDTVKESAKNFAKSVNWV 207
MZ210029 Fushun monoleptalauta Solinvi-like virus 1   IT---SFLKTNLI-----LSVTNLA------MSILGLVSIESEETVKAVINNLCETPEYT 227
KX024775 Nylanderia fulva virus 1                     IK---IALGSAII-----SFVSMLF------QL----F--DYEPT-------------EN 188
KY070327 Diabrotica virgifera virgifera virus 2       TT---L--VVAMV-----SAFVNLV------ST----AILGIAGTVT------AEFPWQQ 218
OQ540582 Apis mellifera solinvivirus-1                -V---G--KVLLI-----NSIVNFI------ST----LLLGIAGVTT------AEFPELP 226
MN918666 Picornavirales [unknown host]                ------------------------------------------------------------ 0
MW251313 Icha Creek insect virus                      LKHWLGAFGLSLI-----QFVLNLL------MI----INVEIEPE------------EVT 138
MZ822083 Apis picorna-like virus-4                    ----FENYDEY-S-----QFFDNLE------KK----RF----DN------------R-- 36
FJ528584 Solenopsis invicta virus-3                   -------MSE-------------------------------------------------- 3
MW314636 Myrmica rubra picorna-like virus             ------------------------------------------------------------ 0
OL569508 Corparats virus-2                            ----SSEVSER-S-----RLFENII------KD----NKLKLEEQ------------SLP 210
```

```
DQ286292 Rosy apple aphid virus                      ------------------------------------------------------------ 0
OP265432 Penaeus vannamei solinvivirus               EVTSKTIQFV-----AACDALANAVRTRGKA----------------------------A 234
MZ210029 Fushun monoleptalauta Solinvi-like virus 1  TFDNFIVD--------------------VKARKFIGHDL---EKLIREARKNDEDF---- 260
KX024775 Nylanderia fulva virus 1                    EISDVKTQFSELLTDEKVDELDGIV---------------AFQEMCLKARYSPDKI---- 229
KY070327 Diabrotica virgifera virgifera virus 2      TLDTLQ----DE----IVEEIGKVDE---KKFVYINEKHKQYVLMCKE-YYKSDAF---K 263
OQ540582 Apis mellifera solinvivirus-1               SFSDDIQQATEL----IIENLEPVKPSPSKREEFIKHVL-------------DHQF---T 266
MN918666 Picornavirales [unknown host]               ------------------------------------------------------------ 0
MW251313 Icha Creek insect virus                     EIKKA------------LEQTSNLVTDPFPKTTYASKE---------------------- 164
MZ822083 Apis picorna-like virus-4                   ----------------------------------------IPQSARYQ-RREDDSDG--Q 53
FJ528584 Solenopsis invicta virus-3                  -----------------------------KTQTFVQNETHVL-DMTSD-FKSDLSLEKVT 32
MW314636 Myrmica rubra picorna-like virus            ------------------------------------------------------------ 0
OL569508 Corparats virus-2                           PHDKP------------C----------SSKDTYYNPEQFILEDTKSQ-FHEDDSINIGK 247

DQ286292 Rosy apple aphid virus                      ------------------------------------------------------------ 0
OP265432 Penaeus vannamei solinvivirus               --------VALHVASFVSHLLPFAVFVCDLA---------GILPEQGLFTFFKEKAT-EF 276
MZ210029 Fushun monoleptalauta Solinvi-like virus 1  -----------------------VVPRSPLSMKVL----------QKRNDKWEFNRSQAL 287
KX024775 Nylanderia fulva virus 1                    ---------QKLYSSF---KVRKNVPYCLPSVNEAQ--------LVVDSKFWT------- 262
KY070327 Diabrotica virgifera virgifera virus 2      LPQ-AKKIFQSIYADFFSRMDMFILPRVDPPFLIVGGKFRAPVSHNSCLVFKAMLKEW-- 320
OQ540582 Apis mellifera solinvivirus-1               TPMQINEYFRANY-----SEFDFLLPPSRAPLKLP-----QPIR-----DAKDSFDNY-- 309
MN918666 Picornavirales [unknown host]               -------------------MNCIVLPANSAPANGT-----LQVK-----DQWM-DFRY-- 28
MW251313 Icha Creek insect virus                     -------EFLNHYHV---AGKDVSLPLNSPALLQRA--LNGQ---TGRRNFRPCFATYS- 208
MZ822083 Apis picorna-like virus-4                   VVINTG---LAPIVS------EDKIPSMTGNFLEDM--IVGVISKFSDNKLLTLSVAMQI 102
FJ528584 Solenopsis invicta virus-3                  SSVEQTDDLVSKIIN------NNDLDIKDLSFLRNL--LLSTLQYLGIAKFVAINITLSI 84
MW314636 Myrmica rubra picorna-like virus            ------------------------------------------------------------ 0
OL569508 Corparats virus-2                           LVIDQNELFENHMEI------REESPYEDPSIINNF--KSIIMEFVSKFNFDAFSVTAIV 299

DQ286292 Rosy apple aphid virus                      ------------------------------------------------------------ 0
OP265432 Penaeus vannamei solinvivirus               FNPKNLS-----------ELSHSATVDTDY--LFNELTSQP-VHQKLDWSNVLKPEYIFR 322
MZ210029 Fushun monoleptalauta Solinvi-like virus 1  LHIRSIMQWKNEEDYYAFILFHDQGMDELSVKYFNKLIENVVVTMHITPKGTAPESF--R 345
KX024775 Nylanderia fulva virus 1                    -------------KEYI-IFYLGLT---ENLYNILKANPQPAPENTFTWEGAEEYSGE-- 303
KY070327 Diabrotica virgifera virgifera virus 2      --P----------IWFI-AAYYGVEE--HDEI--------------ELWNGAKPTTF--- 348
OQ540582 Apis mellifera solinvivirus-1               --P----------DEFL-IIWWNTNP--RYLFSCRSLIKMISPK---------------- 338
MN918666 Picornavirales [unknown host]               --P----------DWFL-AKFWDISY--EAIAKLRFRVNMSSPQRSSSWSEIQPPMPNIR 73
MW251313 Icha Creek insect virus                     -------------DEVL-ALYWQQPV--EVVKRFRKAVKECV---------------NLR 237
MZ822083 Apis picorna-like virus-4                   VSL----------LTYV-FSLWHIK-------KFSKGLLA-------------------- 124
FJ528584 Solenopsis invicta virus-3                  LSI----------LM---LLINSCA-------KF-------------------------- 98
MW314636 Myrmica rubra picorna-like virus            ------------------------------------------------------------ 0
OL569508 Corparats virus-2                           SQL----------LVVF-GLIWSLRR--SRLNKQLKVIII-------------------- 326

DQ286292 Rosy apple aphid virus                      ---------------------------------------------------------MSL 3
OP265432 Penaeus vannamei solinvivirus               AAVVLV---------LSIFAG--------------TSFAKNKNMNA----------GMLY 349
MZ210029 Fushun monoleptalauta Solinvi-like virus 1  EDM------TPTEWRIELFNTAMTSPCIPSTPHVQKFFRKFPILKSNMVTLENAIQSMDF 399
KX024775 Nylanderia fulva virus 1                    --DPPID--------------------------LQT-------------------QMDQF 316
KY070327 Diabrotica virgifera virgifera virus 2      -MDYPEESEQSDG----------------------------------------------- 360
OQ540582 Apis mellifera solinvivirus-1               --PMNEDVRTDDEPK--------------------------------------------- 351
MN918666 Picornavirales [unknown host]               TVPLPSDEDTPDEYK--------------------------------------------- 88
MW251313 Icha Creek insect virus                     DDPKPVKSVVDEAYVKNVYQN------------IRQ-------------------RVDDY 266
MZ822083 Apis picorna-like virus-4                   ------------------------------------------------------------ 124
FJ528584 Solenopsis invicta virus-3                  ------------------------------------------------------------ 98
MW314636 Myrmica rubra picorna-like virus            ------------------------------------------------------------ 0
OL569508 Corparats virus-2                           ------------------------------------------------------------ 326

DQ286292 Rosy apple aphid virus                      SKASDLEQVKPASLWITTY-------------HTIKQL-------IKEWFIKL-VET--- 39
OP265432 Penaeus vannamei solinvivirus               TAINAGTTLASKATTAGSFLICSLFGI---------------------EMPHETLEKK-- 386
MZ210029 Fushun monoleptalauta Solinvi-like virus 1  SLVSL-DEWEYVPDWIHCYLLCESNGWLEKNVSQLTNEDKRNLQKMKDEYYFSARNKKPD 458
KX024775 Nylanderia fulva virus 1                    QIVTV-PSDGNCMFWSMLA-------------SDAKELTMTNLKTLKDSFNALSLTKE-- 360
KY070327 Diabrotica virgifera virgifera virus 2      ----------------------------------------------------N-MGQE-- 365
OQ540582 Apis mellifera solinvivirus-1               ------------STWRELS-------------SDEEVKA------VKDSFKKK-KEQEAA 379
MN918666 Picornavirales [unknown host]               ------------ADWKKDP-------------SKLPKNA----HIPRHAYKKT-LGRR-- 116
MW251313 Icha Creek insect virus                     Q------GVGKDPIYQAII-------------SELGT---------IIYFLVT-VKHE-- 295
MZ822083 Apis picorna-like virus-4                   ------------AITLQIT-------------SVISTF------SVIFGFPLN--RKE-- 149
FJ528584 Solenopsis invicta virus-3                  ------------TRIVNLS-------------SHILNII----TTLGLYFQVS--SME-- 125
MW314636 Myrmica rubra picorna-like virus            ------------------------------------------------------------ 0
OL569508 Corparats virus-2                           ------------SSFVGLC-------------STIL-------YASKLSFPKK-TIKD-- 351

DQ286292 Rosy apple aphid virus                      --------------------------VLDPGEIFFALTAIV-NTLTGIVLSTVTLFR--- 69
OP265432 Penaeus vannamei solinvivirus               --------A---------------------EELETKTTKIT-HSDY-----ATQA----A 407
MZ210029 Fushun monoleptalauta Solinvi-like virus 1  FYWPPETPIINAIVDIVKLDLTIEEGTV-KEEIVKVYHAVE------------------S 499
KX024775 Nylanderia fulva virus 1                    -------EMQIV----------NTYGAWGTSDMLFKWCTHYRRSLY-----LAQMNKEGI 398
KY070327 Diabrotica virgifera virgifera virus 2      ------------------------------KESIERINQLA-DGGD-----ST------- 382
OQ540582 Apis mellifera solinvivirus-1               VKVTMRKPKFDLPKNPF--IRDH-------KQNKDDWEEIA-RGMD-----A-------- 416
MN918666 Picornavirales [unknown host]               --------------QTF--L----------NKVRRHWQKIK-REEP-----RPEIQSEES 144
MW251313 Icha Creek insect virus                     --------------------------SKENAKLLKQYQEV-------------------- 309
MZ822083 Apis picorna-like virus-4                   --------MQDI-ISES--ISSSVNSKF---SLQEHINWLN-VNAR-------------T 181
FJ528584 Solenopsis invicta virus-3                  --------IEEI-TQTF--E--NEFGTYDDDKILSHY-IKI-CNLP-------------N 157
MW314636 Myrmica rubra picorna-like virus            --------MLNL-FKSF-----TNKDNDKIIDLSSHL-EII-QNLN-------------S 31
OL569508 Corparats virus-2                           --------MENI-VRSV--LLSKKEEDVVMNDISSHK-RII-SQFR-------------S 385
```

```
                                                                                               .

DQ286292 Rosy apple aphid virus                          KGTGYLMKIISGVN--------------LLSTFELLLIQIARDRNSFVPEHAREH-AERR 114
OP265432 Penaeus vannamei solinvivirus                   YEDATLKEYIVQCDLALKKCKTPETA-TLKSRFQTIKRDIEAVRTNLSQISAP---MYLQ 463
MZ210029 Fushun monoleptalauta Solinvi-like virus 1      RVDQFVLNYLPVKNVVDIDHLDPEQMLYIESQLSTLLTRIAIGPREVIEYNDK------- 552
KX024775 Nylanderia fulva virus 1                        GHT--VY--FDGG------------MRKTGPPLKIIYYPIQEHFNAFIEKKGN---VPNK 439
KY070327 Diabrotica virgifera virgifera virus 2          GHD--------------------------------TVDEGEKTPAEQKPLDNF------- 403
OQ540582 Apis mellifera solinvivirus-1                   ------EQELATA----------------------ILDSHKVKPEDVKELQAE------- 441
MN918666 Picornavirales [unknown host]                   GED--MYETLANT----------------------AIWNH--EPTGF---SQS------- 168
MW251313 Icha Creek insect virus                         EED--LLSFTATN------------VE--T----YALGTQGLPPKEYTEHLND--TEIAT 347
MZ822083 Apis picorna-like virus-4                       KRD--ILEYLSRN---------------------YLNNRVSLPIVSFHELAKP------- 211
FJ528584 Solenopsis invicta virus-3                      RKD--VYEYISLN---------------------DLKYKIKLPDISFYELKNDILSKNKN 194
MW314636 Myrmica rubra picorna-like virus                RLE--VHNYIYKE---------------------NLFHLVKMPSCTFDELKNN----KNH 64
OL569508 Corparats virus-2                               RLA--VRKYIFDN---------------------KLENSIKLPSCTFMELEDK----FDD 418

DQ286292 Rosy apple aphid virus                          LSEYLDYSDTEE------------V-------------YA------NTEQEAESMWVSSL 143
OP265432 Penaeus vannamei solinvivirus                   MVQWNEYTFPSEKDLNFASWTQDTLE-----------------KCMQFNTFMDSVTAL-I 505
MZ210029 Fushun monoleptalauta Solinvi-like virus 1      ---------------IRIIQQVISFYRYLRAQKLRQMEFHPPMV----KQFFESISPG-L 592
KX024775 Nylanderia fulva virus 1                        GEQWYNGTKIDP---TTTDWAEAANK---QDE----QKPV--EGVNQSDSMKDKFTDL-A 486
KY070327 Diabrotica virgifera virgifera virus 2          ---------TKE---LEITEEDGKTT---------------------AYDEPTRISKI-I 429
OQ540582 Apis mellifera solinvivirus-1                   ---------IKE---IEDALKPKPVE---EPIPLQQENFF-----EDSFVSMDSLKNF-A 480
MN918666 Picornavirales [unknown host]                   ---------VDN---MEFIELPGNTR---RVASLNLDDYVKQDDSDDDEISMKKMWKI-F 212
MW251313 Icha Creek insect virus                         LKQWIRGQHVGK------------------------------MV--REDWFNDSVVPL-V 374
MZ822083 Apis picorna-like virus-4                       ENDWIWNEYSDS---FLAAWYGKTVS---FVLEQRRIPYVRLEK--QT---QHTVMRS-G 259
FJ528584 Solenopsis invicta virus-3                      LHLWIFQKFTDE---FLAMWFGVQPY---RISNLREMLVIS-----RQGFIPKDLFNE-I 242
MW314636 Myrmica rubra picorna-like virus                LLDWVFETFNDK---FLAMWFGVEVY---KLVHLRRANIQLINQ--DFSNMGKETYKM-V 115
OL569508 Corparats virus-2                               NYYWIFKNFDDE---FLAMWFGVALW---KLESIRSKIPVE--L--EKQSFIQDYYVF-L 467

DQ286292 Rosy apple aphid virus                          STILTTLFGALLAGFGLSTKGLE-------GLTTTFRAATTTKNGVFCIKELVDNALKLI 196
OP265432 Penaeus vannamei solinvivirus                   P-----------------------ERHPVSPLYRSLKAKS----------EVVRRILESR 532
MZ210029 Fushun monoleptalauta Solinvi-like virus 1      QKLGAWLIVI-LLSISHKILPKFGLEFDASKFAANYMSFSKLR-------TEATKSFTEI 644
KX024775 Nylanderia fulva virus 1                        VEIFDKIFGL-LFGIIYIK--TTKTNAPLRDLATSFSLGKLLK-------EGAVDTKAAV 536
KY070327 Diabrotica virgifera virgifera virus 2          DIMFSKVIAL-FASITSCL--LEKKKITPRTIITALAEAEKVE-------GVVGDFKEIL 479
OQ540582 Apis mellifera solinvivirus-1                   EKIMIQVGCI-IMAIGDAF--VTKKTLNFKSIVDYLSLSKKIN-------TIGGDLKEAI 530
MN918666 Picornavirales [unknown host]                   KRIGLRITAI-CIYVFDTC--TTKQKFSLAGLVKSLRMVDTLG-------KESTEAQELF 262
MW251313 Icha Creek insect virus                         KKVISMIVGI-ITSILFAK--ATGSKLSSREIVSGISIGKSLA-------DVGNDIEKVI 424
MZ822083 Apis picorna-like virus-4                       IEFAQRFLGI-VLSWLMHL--IDGKPFSVSSVKDGIMIGNTIS-------NCSSDLIKDF 309
FJ528584 Solenopsis invicta virus-3                      RKLCNMGVSV-IISFIQSK--LFDEPFKKRDCTQALKDASVIS-------SPFDTLWNLI 292
MW314636 Myrmica rubra picorna-like virus                KEFLFRAFGI-LTSYVSHK--VSGEKFKASSFMGGIATGKDIA-------SFTGDFFDSV 165
OL569508 Corparats virus-2                               KQLALRLFGI-TTSWILTK--IHGENFTKRDIMDGIQLAKDFN-------TFSGDIIDTI 517

DQ286292 Rosy apple aphid virus                          AEEKYDPVY------KERKAITELTEEAQELLRLNIGAIASDNKVRDRYRTIIKDINLAL 250
OP265432 Penaeus vannamei solinvivirus                   QRWLSE--GGD----LCTQPYDRILAKLDSWLQTTAAAFHTEPSRYHDFDADLNMAQALL 586
MZ210029 Fushun monoleptalauta Solinvi-like virus 1      AELFGMETDKS----KYQKKCADMIELLNSFLAKPAYYYASDVSKISDFKAATETAEAIL 700
KX024775 Nylanderia fulva virus 1                        EKIFLEKDPEK----QFIAQCEAISKKLHLYMEIPPYKMANSATLYNEWKNYQREVETFR 592
KY070327 Diabrotica virgifera virgifera virus 2          KVVKGDPTTDE----NLRKTVTDISTELTKFNEMPTHQFALHPRNTQRFREVIDKAQIVL 535
OQ540582 Apis mellifera solinvivirus-1                   YVLLNKETPGD----SLSNQAAEFALQFNQFLEQPSHVLARSLHLVAKIKDTLGDYERWQ 586
MN918666 Picornavirales [unknown host]                   NLIVGRDTHEK----TFNKKAEEFRTYFNKFMEIPTFEFAATPITVYKAKERCEEFDKWY 318
MW251313 Icha Creek insect virus                         CGKFEDKSEAEAKEQEGKDLITKYCNVCNEISETPPHVFEKQPHKIETIKGQLNEVLNFL 484
MZ822083 Apis picorna-like virus-4                       IDTFDS--DKK----YFEKQVTKYVDDLEKFLETPNYEFSNNLRYVYDISDELKEIRTFI 363
FJ528584 Solenopsis invicta virus-3                      SKQVCDNSAEE----RFTQTILDFTSEFDNFLGIPNYKFAKNQKLVNTISKSLDACAKFI 348
MW314636 Myrmica rubra picorna-like virus                TKNFFK--HDD----PLLDTISKYQKDFDEFNATPTHQFMRNNRKIYDISDKLNACSEFI 219
OL569508 Corparats virus-2                               TGISVN--KRS----FYRIQSEEYVKHFNSFLEIPTYDFVRNQRKIHDINSMLEDCNKFI 571

                                                                                                             RNA helicase
                                                                                                        (P-loop NTPase domain)
                                                                                                             (AmSV1: 630-750)

DQ286292 Rosy apple aphid virus                          TT-YRH---QDSANMRSLLATMAGELNKKLAEVESLIPTRQVTAGVYLQGEKGVGKSSLC 306
OP265432 Penaeus vannamei solinvivirus                   KNPINDHTRHAHSMLTSSYVKA-KQHLAELVK-DAAQASRPPTVVIQLVSEFGHGKSRLT 644
MZ210029 Fushun monoleptalauta Solinvi-like virus 1      AEAITPEFKAISQVMNGVYTSA-NKKRLEITSSTLLSTARFEPIVYCFQGLAGVGKTQLA 759
KX024775 Nylanderia fulva virus 1                        LN-APKEYANTVRSLSSLIDAS-SNLITQIACMQHKSLPRQEPVAVLFTGTGGIGKTQAL 650
KY070327 Diabrotica virgifera virgifera virus 2          NS-MEPKIRAKFPLLQTAITTS-TVRKNELLTTELPSLQRQEPFLVLLRGEPFVGKTELS 593
OQ540582 Apis mellifera solinvivirus-1                   RA-LKNEDQHKFTGLHGLYAAA-SKKRFNFITSEMPTLSRQEPFVVLLQGGTGVGKTNLA 644
MN918666 Picornavirales [unknown host]                   HG-LVQEEKQKIASVYTLNATT-RTRIQQFFQTEIKQMARQEPFLILIQGPGGIGKTQLA 376
MW251313 Icha Creek insect virus                         NE-QPKDQQSNLIALRGLYQAA-NIFKNQLLTNKMPSFVRQEPFIVCLSGPSGIGKSQLA 542
MZ822083 Apis picorna-like virus-4                       SD-YPKEQQQQIIPLMSIFAAA-SKRKNEILTSMSPEFHRQEPFLILLRGPGAIGKTYFA 421
FJ528584 Solenopsis invicta virus-3                      RD-CPKDKQTEIFPLQGLHTAT-VKRRNEILTNVMPKFARQEPFVVLFQGPGGIGKTHLV 406
MW314636 Myrmica rubra picorna-like virus                RS-VPRDRQHELFPLQGLYNSV-CQRRNDILQNAIPEFKRQEPFCVLVTGKGGIGKTRLI 277
OL569508 Corparats virus-2                               RE-APKEYKDLLYPLQGLSTAV-YKRKNELLTSSIPNFKRQEPFVVLFQGQGGIGKTSFI 629
                                                                       :             .:         *       . .    **:

DQ286292 Rosy apple aphid virus                          NYLASYLAEVF----DWSPAVYNISPGVRFYEPYLNED-FGVLNEFGS--TVDRDAFSVD 359
OP265432 Penaeus vannamei solinvivirus                   TDHLIPDVANLLGI-SPSTYFINI-SENGHWSRYLGQN-FAICDEYLR--CMSKDALFKH 699
MZ210029 Fushun monoleptalauta Solinvi-like virus 1      LELAKQLGEKLFPHVPSETRIIHVTSVDKYWPRLNGQP-IAFFDESASTADLERCLLWRN 818
KX024775 Nylanderia fulva virus 1                        MNLAYKVKEKLYPGMTYHEAIGTYPPSQKYWPTLHGEP-IGIYDEVASCTTFADEPLLRD 709
KY070327 Diabrotica virgifera virgifera virus 2          LQLSRWFAKEILQK-DYHGSFVQITPSDKYWPPLGGQE-IALFDDAATNKDLRTDLLFSN 651
OQ540582 Apis mellifera solinvivirus-1                   DEIVYRAARDILRV-SPASAAVDITPSDKYWPPLGGMKDIYKFDEACTTKDIEKDLLFGN 703
MN918666 Picornavirales [unknown host]                   KHLARKVCTEILKC-DPNDNIAEVTPTDKYWPPSGGHA-VAIFDEAASAKDLREDLLFKN 434
MW251313 Icha Creek insect virus                         NYLIKFIDTSVLRQIEGLNNVISINSQDKYFPPLSGQR-IAFFDEAGTVKDFSKDLLFGN 601
MZ822083 Apis picorna-like virus-4                       QHLAKRIVKELIPDGDLKRDYIEVRHDDKYWPPLAGQR-VAFFDEAGTVRNIADDLLFSN 480
FJ528584 Solenopsis invicta virus-3                      QQLATKCVNSFYQDH--EDDYIEISPDDKYWPPLSGQR-VAFFDEAGNLNDLTEDLLFRN 463
MW314636 Myrmica rubra picorna-like virus                QDYLSIETLKFMDNATSAHDYIEIRADDKYWPPLSGQR-VAFFDEAGTYQNIENDLLFRN 336
OL569508 Corparats virus-2                               QQLAGKCTTKLINDGSMDKDYIEIRHDDKYWPPLAGQR-VAFFDEAGTVADIKDDLLFSN 688
                                                                   .                  .:    .   .   ::      .    :  .
```

```
DQ286292 Rosy apple aphid virus                      ANRICSSDPFNFESAGLSGKVSMCQLKALFVTTNVLNPDIKELM-----PNAVSAFWDRF 414
OP265432 Penaeus vannamei solinvivirus               IAEIASNARFDVNSAEIAGKGVPFQSKVLFLNSNQTRCTFPELS-----PDHVDAIHSRI 754
MZ210029 Fushun monoleptalauta Solinvi-like virus 1  IRALASPASFNCEAAAVDLKMQPCPFKVIITTTNTQLKELTRLIAEYNGNDAIVPAFSRV 878
KX024775 Nylanderia fulva virus 1                    IKSIVSAVHFNCEGAALSQKQNPMQQHLICMSSNQTLEGLQKMASDQFDKSSVPSFWRRI 769
KY070327 Diabrotica virgifera virgifera virus 2      LKGICSPAYFNASAADIAHKINPIHFQVIFATINSTFDNLAARVTEFSDNVTWAAMCRRA 711
OQ540582 Apis mellifera solinvivirus-1               MKGICSPAYFNCAGADVEHKTNPCTAKIIVATSNTDFSDMAARVSEKTDPTTWQAYCRRM 763
MN918666 Picornavirales [unknown host]               LKSICSPVLFNAAGADIAHKLNPIFYKVIIATMNTSLQNFSDRIVSMYDQESMDACARRI 494
MW251313 Icha Creek insect virus                     IKGICSRNFFNAAAADIIHKTNPTYFEVIIAATNTTPDDLVKRYGAITDSSSIPAMYRRM 661
MZ822083 Apis picorna-like virus-4                   IKGLCSTAYFNCPAADVEHKVTPCPFHVIIATTNSELDDIQGKYGAFAGPASVYSMWRRL 540
FJ528584 Solenopsis invicta virus-3                  IKSICSPAYFNCAAADIEHKISPCPFELVFATVNTDLDTLQSKISSTFGQASVFPIWRRC 523
MW314636 Myrmica rubra picorna-like virus            MQSICSSAYFNCPTAALTHKVTPCPFQVVYATTNTDLYTLQGRVSSGMSANSVYPFWRRC 396
OL569508 Corparats virus-2                           IKGICSPAYFNMPAADIEHKVIPCPFHLIFASTNRKLFDLQAKLSLAFSSDSVYPIWRRC 748
                                                        : *   *:   * :  *      . :    *     :                  *

DQ286292 Rosy apple aphid virus                      IIIKVIDPLVTDRRK------------Q-VNHREPDFSHLSFKIYELHSDEGELPKFKML 461
OP265432 Penaeus vannamei solinvivirus               MTIQVFNPEWAKY---VADCKQRGTVPIRTHPVNRDPKRVKFALCSFGTANE-IVSKKPL 810
MZ210029 Fushun monoleptalauta Solinvi-like virus 1  RVY---SCSRTEA-YGPVDLNDRNS--I-RYDSTGKFRHLTLHRMKIDSQESRQFVADQL 931
KX024775 Nylanderia fulva virus 1                    QTY---ECARPDN-MPPFDP---NN-PQ-PGDFRKDYSHIYFNKHVFDVKTGTTRFLQRL 820
KY070327 Diabrotica virgifera virgifera virus 2      IVI---DMTWKDQKM-VGNLSKDSTLGK-DKPHKKNFSHANLSLQRMD--GGKLQHYKHI 764
OQ540582 Apis mellifera solinvivirus-1               III---QVER--VSK-HFD---PNYPEQ-DTFDAENYSHLKLSQFEHR--DGKLIKTKQL 811
MN918666 Picornavirales [unknown host]               IVL---TTEWNPKAG-HHN---PNEPWS-NAYDKKNFTHLNIKLQKKN--KGVYVDDKPV 544
MW251313 Icha Creek insect virus                     IFI---NVSHVNENFDPTRC-------S-EQDYKSDFSHLRLNKINLDIGSNKVSLGNYV 710
MZ822083 Apis picorna-like virus-4                   LVV---DCKWAGE--TKLNL------ND-PTGHRSDFSHIEFTRCEWDDSKSRMVKTETI 588
FJ528584 Solenopsis invicta virus-3                  IVV---ECSWNEKELGPFNY------KN-PSGHRSDYSHITMNYMSYDDKTQKLALEKEI 573
MW314636 Myrmica rubra picorna-like virus            LAI---EVEWNADLMGEFNY------RE-PTGHRSDMKHLRIKLMEWDQNQGRMVFSKNL 446
OL569508 Corparats virus-2                           IVI---TTEWNEQRFGKFNF------VN-PQGYQSDFSHLKFKINQWNQQTQRLEEASDI 798
                                                                                        .  :  :               . :

DQ286292 Rosy apple aphid virus                      -YEKL--------------------HINDILQILRTK----LALETRAF---YHRLNHPM 493
OP265432 Penaeus vannamei solinvivirus               DYDNLVKFIARLIQDNDTQRSTALTTIDDLADQITQVTPAPVNYQFRPDMSRQGVAEFPD 870
MZ210029 Fushun monoleptalauta Solinvi-like virus 1  -----------------------STSFDQLLTEALA------WYDIKEI-------EH-- 953
KX024775 Nylanderia fulva virus 1                    -------------------------TMDEVIADTVS------RIQTKKN--R-HQVEM-- 844
KY070327 Diabrotica virgifera virgifera virus 2      -------------------------SVSQLKTLIQD------AHRHECG-------EF-- 784
OQ540582 Apis mellifera solinvivirus-1               -------------------------DLKRLMRMIED------KENFMAN-------KH-- 831
MN918666 Picornavirales [unknown host]               -------------------------QVKDIMEMAKQ------REAHFSK-------DF-- 564
MW251313 Icha Creek insect virus                     -------------------------SIAALKDMIVD------RYGKAAT-------EF-- 730
MZ822083 Apis picorna-like virus-4                   -------------------------GPEKLFNIAAK------MYQKRYI-------EH-- 608
FJ528584 Solenopsis invicta virus-3                  -------------------------NFDTLFDMIRL------RFRKKQQ-------EH-- 593
MW314636 Myrmica rubra picorna-like virus            -------------------------SREELLEMIKQ------RYRKMEA-------EH-- 466
OL569508 Corparats virus-2                           -------------------------SVEDIYELIRN------RYRKMES-------DH-- 818
                                                                                  :                          .

DQ286292 Rosy apple aphid virus                      PEVLQKIVEAETNPPV--GSYAFKYF------------------GFQEKVEENSSGRDFC 533
OP265432 Penaeus vannamei solinvivirus               LRPVTM-IEA-KNKTVKNGALSCIFCKGGHLSETCTQFVQGPEHAPRWQAGPVDSCVNHL 928
MZ210029 Fushun monoleptalauta Solinvi-like virus 1  -----------------AALLRKNFY---------------------AKEKQSKTARLHL 975
KX024775 Nylanderia fulva virus 1                    -----------------DKLLSET-A---------------------ISGVNQSDSADHF 865
KY070327 Diabrotica virgifera virgifera virus 2      -----------------ERRIRLD-Q---------------------CIQESEPASRTHF 805
OQ540582 Apis mellifera solinvivirus-1               -----------------RAHLA-K------------------------MALVKENSITHY 849
MN918666 Picornavirales [unknown host]               -----------------TNWLHLK------------------------NQKPESGSKSHY 583
MW251313 Icha Creek insect virus                     -----------------DQN--CL-N---------------------SMIKESNTNVQHF 749
MZ822083 Apis picorna-like virus-4                   -----------------KRNIDLQ-R-----------------------VEKQAEHRMHY 627
FJ528584 Solenopsis invicta virus-3                  -----------------DTKISIL-N---------------------NEIQRQSNSKQHF 614
MW314636 Myrmica rubra picorna-like virus            -----------------VRFLNMQ-G----------------------LVKQV-DQRIHY 485
OL569508 Corparats virus-2                           -----------------SRVIDNM-N----------------------MLKQSNLHKSHY 838
                                                                                                               .

DQ286292 Rosy apple aphid virus                      ILRLQGPEGTGKTTL-AMKLAEKWTELTTAFEGKSRYKIQKSSNLQEFVPSDTSTIYVLD 592
OP265432 Penaeus vannamei solinvivirus               VFHLYGKPGFGKSHTTINHIVPALQTLTKL----HSVKLDSFDEV-----FETPMIVFID 979
MZ210029 Fushun monoleptalauta Solinvi-like virus 1  SVCIFGLPGGGKSTL-VEKILPDLSVATTL----PIVNLSEDTII-----PKHKCIFVVD 1025
KX024775 Nylanderia fulva virus 1                    VVNINGRAAAGKSRL-LEAAVSDFVKVLNY----QVVRYCDLEKMAGGKRLKKRVILIVD 920
KY070327 Diabrotica virgifera virgifera virus 2      SVVIHGRTGQGKTYT-LEQQLANLTTAFAY----PLIRINNVYDIKNLPVQRSRAIVVMD 860
OQ540582 Apis mellifera solinvivirus-1               SMVIHGQQGQGKSYF-IEYLIRNMPSAFNF----PIHYIKEKSDLLKFRKQTKRCILCID 904
MN918666 Picornavirales [unknown host]               SVVFHGRAGQGKSFA-AMESFVRLSESLHF----DGFVAKSFAEL-SKLVFKKRCVLLLD 637
MW251313 Icha Creek insect virus                     SVVMHGAGGHGKTPL-IGQISNNLSKVLRL----PILKLFSENDINNLKTQNSRSIVVLD 804
MZ822083 Apis picorna-like virus-4                   TLHMHGPPGQGKTPA-LLNLASKLSSLYSM----PVFVIKELRDFQQLRFKDNPSIFVFD 682
FJ528584 Solenopsis invicta virus-3                  SVCLYGEPGQGKTYN-LNKLITT---------------FANATNLKI-GSEEKPSIHIFD 657
MW314636 Myrmica rubra picorna-like virus            SCHLRGPPSAGKTPQ-LEQISKQLSINFKY----PIINFEEFNQLKQ-T-YDFRIIAVFD 538
OL569508 Corparats virus-2                           CVHLHGSPGQGKTPS-ILEYAKSLKRIYSM----PVKIFTSWDEVKN-HHSNTRSIYILD 892
                                                        : *  . **:                           .   .          :  .*

DQ286292 Rosy apple aphid virus                      DWVSSNLTEQESSAYVTKMNATNNDSLFIITTNEVIKRRLPWCELPTRIIMGL-IGNPYN 651
OP265432 Penaeus vannamei solinvivirus               DMVLD-----DQAGYLRWWSAQP-VSHIVVLCSNMKAKTDKWKTRKDYFRRALSKSVEDS 1033
MZ210029 Fushun monoleptalauta Solinvi-like virus 1  DYLPVQADPIREKQFMDLYNNQMENNSIIIACTNKIPWRIPTGVTFTVLE-GFTIPVPYI 1084
KX024775 Nylanderia fulva virus 1                    DDIF-LNSPEIEQKYMHCYNNILANGSVIFVATNISPGYSR----IPVIG-T-----NGY 969
KY070327 Diabrotica virgifera virgifera virus 2      DVVKENPSSELQEAIMEMFNTKLVHASIIVTVTNVSPKFQ-----SFTLT-G-----RDF 909
OQ540582 Apis mellifera solinvivirus-1               DVIRNTADPEIENAFMDIYNNRLAPCSIVVNATNVSPKPT-----IPYLG-G-----THF 953
MN918666 Picornavirales [unknown host]               DLLRETMTAEEENILMDLYNERLHDSSVVIFTTNVSPKRT-----IPIFD-A-----LSI 686
MW251313 Icha Creek insect virus                     DVIKIGSDLSLEKSLMDLYNNKLYPNSIILSATNLSPKYSA----FKF-T-T-----KGI 853
MZ822083 Apis picorna-like virus-4                   DIFDNNCSVELQHEFMNIYNNIMPNNSIVLVSSNISTGYSM----KPYLN-L-----TNI 732
FJ528584 Solenopsis invicta virus-3                  DYI-KDENDENCSKFMDIYNNKLPNNSVIFSATNVYPKTHF----FPTFF-L-----TNL 706
MW314636 Myrmica rubra picorna-like virus            DIFHSRNTAEELDLFMKFYNLRCADGSIFLFASNLPNNYSR----IPKVS-Q-----LTL 588
OL569508 Corparats virus-2                           DMFINQLSSENQQIYLDWYNNRIIDSSIILICSNLKIKYS-----LPIIR-G-----TNL 941
                                                     * .            :   .       ...  .:
```

```
DQ286292 Rosy apple aphid virus                      YPYDMNPFKGLYPGIRRRIGIDDLYSVNGKVHSVTSTEYCMVVTTDEKCVLTAKGKN-VG 710
OP265432 Penaeus vannamei solinvivirus               RFFKIKEL--KVPGVVRRLGINGYVKFDDNW-----ESTQS-----GRCFHVKSGCI--T 1079
MZ210029 Fushun monoleptalauta Solinvi-like virus 1  KWQQYLPT--YEPGLIRRYGWQGDFLFS-DTNLGNLNILCD-----NRTYYQNVQQQ-LT 1135
KX024775 Nylanderia fulva virus 1                    KIIRTSPF--INEGMVRRMGYIGSFVDT-GSSKHGIEFIAH-----EELYHIPKDQV-IQ 1020
KY070327 Diabrotica virgifera virgifera virus 2      TISRKLPF--QNEGVARRLGYKGVINGI-VMPTANIELHVE-----NFRFHVVQGSLGFR 961
OQ540582 Apis mellifera solinvivirus-1               CANRKLPF--TNEGMGRRMGYVGCMDGN-YLPTFNTEIYVS-----NQTYYKRDSNITLP 1005
MN918666 Picornavirales [unknown host]               VPQRSLPF--VNEGLIRRAGFKGYYHGA-LAPSTGLEIRCN-----NMRYYSFDQVRSIL 738
MW251313 Icha Creek insect virus                     LLLKNHPF--VNIGVTRRLGFMGAFTDG-YTPNYNLDYKVV-----KSKLIPERDFI-IP 904
MZ822083 Apis picorna-like virus-4                   YFPRTLKF--KVTGIIRRLGFCGRFDGLGDMPGYNREYYCL-----NNSFFRCSPKP-IN 784
FJ528584 Solenopsis invicta virus-3                  IYAFIQPF--KQVGLYRRLGFDGYTDIP--NSSVNAPIFVQ-----NFKFYERKQH---- 753
MW314636 Myrmica rubra picorna-like virus            AIDRRHPY--DLPGITRRIGLRGNFNGE-VMPSYNDEIFFN-----NEQGTEPIVYT-IP 639
OL569508 Corparats virus-2                           IIPRKHPF--ELNSLTRRIGLTGRFANE-LVPNYNVEFIFK-----NDQAYIRDEIS-FP 992
                                                                  .: ** *  .

DQ286292 Rosy apple aphid virus                      F----------------------------------------------------------- 711
OP265432 Penaeus vannamei solinvivirus               TMDD------QLI-DLDKEIQDFA-----------VLWHKFTNTVYTILP---------- 1111
MZ210029 Fushun monoleptalauta Solinvi-like virus 1  LYDY----LKFAIPLLTGIFTCFY---SLILGFSLIIL-----AVYF------------- 1170
KX024775 Nylanderia fulva virus 1                    FPDF-----SPKHEITFGNLMNLNTTNQTFFD---MCKGAAKFATFTAKQAIMGPGTQLA 1072
KY070327 Diabrotica virgifera virgifera virus 2      IRYLI-TLIPILIAVLMAN------------FYSLLFAAIYTVGLFIVPV---------- 998
OQ540582 Apis mellifera solinvivirus-1               FSTFIYAVLPFVISVSCILF-------HPIFFHISILLGIY--AMYKTKI---------- 1046
MN918666 Picornavirales [unknown host]               IANI--SVFNWIIIVLASLV-------QMYFIYLPVFTIVY--AYY-------------- 773
MW251313 Icha Creek insect virus                     YYYF----LPILA-----TIIDYR------IIVPSIILVCALISCI-------------- 935
MZ822083 Apis picorna-like virus-4                   R--F----LLFLAFISLP----LSFW-NAYFL--FMCLPLF-----F------------- 813
FJ528584 Solenopsis invicta virus-3                  ---I----CYFLSLEFLKNI---------------ICYIFFFL--YF--P---------- 777
MW314636 Myrmica rubra picorna-like virus            TMEV----VIPLSILLFGMLMSYSFIPNYYFIPHFINIIELFITCYV--I---------- 683
OL569508 Corparats virus-2                           WWKI----LLFMLSLMSGLI-------NPIFIVSLPVLVFFLNSC--------------- 1026

DQ286292 Rosy apple aphid virus                      ----------------PYIVENIKKRFISMIENRYNIVTIQGRPENELPTVDFEIKAANK 755
OP265432 Penaeus vannamei solinvivirus               ----------SNDIPNKADIMLMFDSYDEAVKAFSSTM--------------SVLKAASK 1147
MZ210029 Fushun monoleptalauta Solinvi-like virus 1  -----SSQQKLKQIQVGAMSSLVYKNYASLISNGADIGVKIQSVP-DRPNAMINFYAESI 1224
KX024775 Nylanderia fulva virus 1                    AGYSPALAFNMASYTRADAKDMIYKQYMKWLDSRKDYLVVYEPV--PTVDWNFKIFANRA 1130
KY070327 Diabrotica virgifera virgifera virus 2      ----CFPEKYEQELTPDQLPNYVYTKYGAFIEEIKTVSVQH-GLVTKPDDCNFEIYAKSS 1053
OQ540582 Apis mellifera solinvivirus-1               ----LWTQDKQQGFPCNDLAQYVFQCYRDFMSTQREIPIHYRTF-TKMDDYNLLIKARTG 1101
MN918666 Picornavirales [unknown host]               -----YLGNTEIEIRLEDLVPAFYEGYKAYLTAKKDVAVRYVNLIERTGTYNLLIDSESA 828
MW251313 Icha Creek insect virus                     ----NHFTKFKHSICSGDIEKVVLKSYQNFNELHTGLKTI-YDQE-LTGDFNMILMAERA 989
MZ822083 Apis picorna-like virus-4                   -----SLRRLHDRMLVNDIVEDAYSSYKEFVRMRHDIPINIVD-ELPNAEYNFCIKIPKP 867
FJ528584 Solenopsis invicta virus-3                  ----LKFIKKIDLIEIKDVNKYVYDRYINFLSLSKQIEIVEYPPNLENVEFDFRFNMNKF 833
MW314636 Myrmica rubra picorna-like virus            ----KKRNKLFTKTLPKNLNEYVYNRYMAFIKSRDDFVVREGEIP--DSDFSYQLEADSI 737
OL569508 Corparats virus-2                           -----------KNSQISDPEQYIFDRYSDFIKMKQQCTISEGSRD-TIGEYDYILQSESL 1074
                                                                               :                           :

DQ286292 Rosy apple aphid virus                      DILF-----KKLSTSLG------AASLYLKPDAEASLYVSERVISGIAEVNPDKAIRPSD 804
OP265432 Penaeus vannamei solinvivirus               GKIVVRPGLSGNTANVDLSDMKLPE-EYPISNLEWTRFASV--VSRVYTSARSVFVSI-- 1202
MZ210029 Fushun monoleptalauta Solinvi-like virus 1  KNIK----IPADVISLQPHIFGSVE-TFDKAVVPWKIRIDSNILSVVLPNMSKFLVSQQ- 1278
KX024775 Nylanderia fulva virus 1                    CDVR----LSHNYLEMINNIFYDPV-KFDKCDAPWKLFITRDVFDACMKDKSKFSINVS- 1184
KY070327 Diabrotica virgifera virgifera virus 2      DVVE----FKADPYEMEKHVLYDRA-RYEACDLDWKLYLDKAVFEALMDNYKKFMIEMK- 1107
OQ540582 Apis mellifera solinvivirus-1               DDVI----MSGDIADLSKHVFCNKS-LYDRCRLPWKLYISPEIFCSFSKNYEKFLMRCN- 1155
MN918666 Picornavirales [unknown host]               NCFM----KYNTISELDKHIFLNSE-EFDRSVANWKIHLPLSVCRLIQQNLERYLLSSY- 882
MW251313 Icha Creek insect virus                     SDIK----ITTNPFDYIDNVFYSKE-KFDNSNPGWKFFLDYNLYKKMYDHSENFLINID- 1043
MZ822083 Apis picorna-like virus-4                   QEFT----LPRNILQLDKHVTISKQ-VYDDSHDDWKMYFSNDIAKKLRCVYHKFFINDT- 921
FJ528584 Solenopsis invicta virus-3                  HRVS----FNNPF-ELDKYIHFNKNSYENLLHFDWKMYLSPRVKHRLALSYEKFFITIS- 887
MW314636 Myrmica rubra picorna-like virus            KSVD----FSKLDCSTLQKIYTNKLSFKNADTKPWKLYISDRLTTAFRDTYQQFEVNCS- 792
OL569508 Corparats virus-2                           EKLN----FSTDKISNIRHLHS-KLHEFDKDENSWKMYVTQNLIETFKNNYELFLIDCS- 1128
                                                       .           .                    .                   :

DQ286292 Rosy apple aphid virus                      FICKEVFDETIPTEERYTTIVERVSRVLLQYFKDP-TFRV-------VIQDE-KRVVYSE 855
OP265432 Penaeus vannamei solinvivirus               ------------GDDIVFADL-AMRKILKNSNLSPVQIFTSRDKKQVIIRDASGKRYTYD 1249
MZ210029 Fushun monoleptalauta Solinvi-like virus 1  ----------VKTREDVLRMIKSYVNMLQSLNVNDIVISV-------VIRDL-G-KFLYE 1319
KX024775 Nylanderia fulva virus 1                    ----------NFTEDVILNVVRKYVDGLTALGIEP-KLLA-------DIGDQ-G-VYAFV 1224
KY070327 Diabrotica virgifera virgifera virus 2      ----------GLDYDKILAIVQRYVMGFRQIGIAP-RFYV-------QIDGT-G-KFSFL 1147
OQ540582 Apis mellifera solinvivirus-1               ----------GFSEELMYTIVRKYVNALKDSGIEP-KFYV-------EIDDL-G-RLEFR 1195
MN918666 Picornavirales [unknown host]               ----------NFDEESIISLIKRYSGAFAMSGLEP-RFYA-------EFRDI-G-RFQFE 922
MW251313 Icha Creek insect virus                     ----------EFNTDTIFSLVQQYAMEFAKLGIPA-RFKV-------HVGDL-G-KFYLE 1083
MZ822083 Apis picorna-like virus-4                   ----------NLSDEVVTRELRRCARSMLEYGVQP-VMLI-------EINNL-G-RYAFF 961
FJ528584 Solenopsis invicta virus-3                  ----------EVNKEIIIEELKRYVLLFKQFNIDP-NMEI-------NLGEY-G-SFYYI 927
MW314636 Myrmica rubra picorna-like virus            ----------GFDYLACIEELKRLARIMRSSNINP-NIRV-------FFKDV-G-EFSCV 832
OL569508 Corparats virus-2                           ----------QFSHDHILKELQRIARIFIQYGISP-KIKI-------IVKNL-G-EYYLK 1168
                                                                         :      :         :          .

DQ286292 Rosy apple aphid virus                      NGVVYYYEDKEDTR----------ISFEGYNLIINVSTPKMVFPYDFAMALNE--DRIEK 903
OP265432 Penaeus vannamei solinvivirus               SEKFITRVTTTL----------------------------------QDVSYWQ---GLPL 1272
MZ210029 Fushun monoleptalauta Solinvi-like virus 1  QGVLYMDVRQTQNNTVITPQWQAIVITNENADV------LRRYDYDSFFN--AKFKNIPL 1371
KX024775 Nylanderia fulva virus 1                    NGKLHVQKERVVNRYCTFSIKDD-------EVWFEMPNRVIMCKWNSILGYHNGEHSIPI 1277
KY070327 Diabrotica virgifera virgifera virus 2      GNCVMAEVFNPAKNECLVHDTPE-----GT-RVAIMEEMQWKFVDPRDVIYNP---QHAF 1198
OQ540582 Apis mellifera solinvivirus-1               DGEISSDLRTETPA-LLN-FA--IVKTELEYLMLFGE---HTVPVARMIK-EP---ELAE 1244
MN918666 Picornavirales [unknown host]               NGVLSISKFNEGNEHQLN-LTPRIHVFEGEPRIFFMD---KVYNVRQLNGYTA---DDAF 975
MW251313 Icha Creek insect virus                     NNNFYINLNT---------HTNSNITVHQDRILHYSDNVVTECLFTDVMD-YA---TWVS 1130
MZ822083 Apis picorna-like virus-4                   QGAIYKQYKNDS-----NNDNDIAIFCSHTSIVLNSTNRILEIPIHEVFG-NI---NVAI 1012
FJ528584 Solenopsis invicta virus-3                  NGKIHLMTINIESNV--SEI---PVFTDGDYVY-I---SEHKIPVIDLFD-NI---NINS 974
MW314636 Myrmica rubra picorna-like virus            DNLINYKLINKI-----NKTSNYSIAHTANSVLIYGRCPTINIPLDEIFAKTV---NIAT 884
OL569508 Corparats virus-2                           EDSIVYHTYESISRV--DTASDLYLATNHNGLIFIDNNCRVDISFEDVYL-CS---NIPL 1222
```

```
DQ286292 Rosy apple aphid virus                       KFVGTLSTLTITEYTIIEKYYKRAIL-NK-----VCNPFLVAVATRELQL----KRMKDP 953
OP265432 Penaeus vannamei solinvivirus                EA-----------IIYL---MKNHQVIIEEHFPLTFLK-LKKKARRDRNM-------QIV 1310
MZ210029 Fushun monoleptalauta Solinvi-like virus 1  AN---ITKDDLETARIIQEAVRDQKIETDP----AYIHNLNKWNKKETKTVLTVEALKLK 1424
KX024775 Nylanderia fulva virus 1                     EH-----NLNLQETEIFV-----NQFQQLK-ATQEFVNRLTQNVSGNLFEKGVQRAVAVK 1326
KY070327 Diabrotica virgifera virgifera virus 2      DN----SKMQLTSVIAIREFVKTPRFLNHP----SIKILLKNWQQEEIQKDLAMVGKSFA 1250
OQ540582 Apis mellifera solinvivirus-1                TL-----NLSYNESIEYNNYIKSDTFLSSE----AYIQTHKVVVEEEIASFAAAKWKLTL 1295
MN918666 Picornavirales [unknown host]                KD-----GLTFDESAILFKYRTSSEFLLHP----NVVKETRQIAKEDAKVEAALIAKKMK 1026
MW251313 Icha Creek insect virus                      TN-----KLNYATAREISTFIDSIQFAQNP----HFIAFIKNYRRQKMNDQLLARGIEVK 1181
MZ822083 Apis picorna-like virus-4                    KY-----ELTLLEALEYSKFVKSSKFLSLD----HIRKFLVEKQKQDFQLDWSMRYKKLT 1063
FJ528584 Solenopsis invicta virus-3                   KY-----NLSFDQSIALNSFKTGDSFYSNA----KVRKSLSKFVLLNYQTKFKLYLKEAK 1025
MW314636 Myrmica rubra picorna-like virus             KY-----KLNYEQIREFVTYIESSEFVNNP----MITKLRHNFYSQQCTRKQQQKFKELK 935
OL569508 Corparats virus-2                            KY-----KLDIRQSKLIYAYIDSNQFQNSV----DYKNFLNNRYQQISFLTFQNIREKTK 1273
```

```
DQ286292 Rosy apple aphid virus                       SYVRGLMRLQNNYLLKLGLCGLAGVGSVALLYGVYKLIVSTMKQEKEKITDNAFANVGST 1013
OP265432 Penaeus vannamei solinvivirus                QQITNSKAFQVGF------PIAVIGVAATAIFGIAKL-SS--KKK--------EES---- 1349
MZ210029 Fushun monoleptalauta Solinvi-like virus 1  DKFTEFSGTMVGKVFLSLVGVLALFFVTKGVFGLFGSNETAL-------QKGRKEF---- 1473
KX024775 Nylanderia fulva virus 1                     EKVSLFLETPQGKIVRFLLYIFLVSGIIAVVYKLVKSFGN-DKMEEQ-VAKGARKV---- 1380
KY070327 Diabrotica virgifera virgifera virus 2      ERYRSFKEHPLGRVVMILFTIWVGLKIIRKLTSGLGILFGNKSKR---RRQARKQV---- 1303
OQ540582 Apis mellifera solinvivirus-1                DTIKKWKSHPIFKIVLIIFTIVATATIIIRIISFIKG-NSMEKQR---RKGSKKGN---- 1347
MN918666 Picornavirales [unknown host]                DQFMAFVAHPLGLITIVIVGIITSVVLLKAISAAFSGDDEQVPEK---RRTGRKAS---- 1079
MW251313 Icha Creek insect virus                      QTIEGFLSGPYGKIVKVLAAILASIAVITAIVAIV------------------------- 1216
MZ822083 Apis picorna-like virus-4                    DKVEEIKRQPFGKFLICLYSILALFLVSAVAYKIFGYFFKTRKRVNN-IC---------- 1112
FJ528584 Solenopsis invicta virus-3                   DKVKNFIETPIGHLLSILLTIFVICYASFKIYSKFSNFFSKDQAIED-QRKGEKKI---- 1080
MW314636 Myrmica rubra picorna-like virus             DKLTNFFITPTGFFIRSLMEIIITLITTIL---------------------IMKMV---- 970
OL569508 Corparats virus-2                            RLLIKFFGTPEGRLGEFIVGMIFFLFSMYRVFNGISSLFKSKKVQDD-PELEAQIF---- 1328
```

```
DQ286292 Rosy apple aphid virus                       PPKRAMNSQSKKDRKRLGVTLTEPIIENSPHQ---K----------HR------------ 1048
OP265432 Penaeus vannamei solinvivirus                -PKSK----------P------------------SHHDSDS------------------- 1361
MZ210029 Fushun monoleptalauta Solinvi-like virus 1  -IAKFHGKLSKNGQRPSDLLLIDPEFEICSECSNNNWNSCVHICEKHPERQWVCYNCENK 1532
KX024775 Nylanderia fulva virus 1                     -RAS------------------------------KKANTDE------D------------ 1391
KY070327 Diabrotica virgifera virgifera virus 2      -VES------------------------------ETSS---------------------- 1310
OQ540582 Apis mellifera solinvivirus-1                -PKS------------------------------KKYDSDD------DH----------- 1359
MN918666 Picornavirales [unknown host]                -PKS------------------------------RNYDSDD------DR----------- 1091
MW251313 Icha Creek insect virus                      -----------------------------------KYFNED------N------------ 1223
MZ822083 Apis picorna-like virus-4                    -----------------------------------D---T-------------------- 1114
FJ528584 Solenopsis invicta virus-3                   -KKI------------------------------TNYDSDG------------------- 1090
MW314636 Myrmica rubra picorna-like virus             -IKV------------------------------INWFNTD------N------------ 981
OL569508 Corparats virus-2                            -AKV------------------------------KTWNKEK------RDQFWSIWANRE- 1350
```

```
DQ286292 Rosy apple aphid virus                       ------------------------------------------GRII--------PNKKRV 1058
OP265432 Penaeus vannamei solinvivirus                ------------------------------------------------------------ 1361
MZ210029 Fushun monoleptalauta Solinvi-like virus 1  VNWTATPISCEYSCSWQYFMMHVLVYYNVCDDDYDKYFTDFC-RMCQGEQTSQ------- 1584
KX024775 Nylanderia fulva virus 1                     ------------------------------------------------------------ 1391
KY070327 Diabrotica virgifera virgifera virus 2      ------------------------------------------------------------ 1310
OQ540582 Apis mellifera solinvivirus-1                ------------------------------------------------------------ 1359
MN918666 Picornavirales [unknown host]                ------------------------------------------------------------ 1091
MW251313 Icha Creek insect virus                      ------------------------------------------------------------ 1223
MZ822083 Apis picorna-like virus-4                    ------------------------------------------------------------ 1114
FJ528584 Solenopsis invicta virus-3                   ------------------------------------------------------------ 1090
MW314636 Myrmica rubra picorna-like virus             --FKDK----DMII---------------------------------------------- 989
OL569508 Corparats virus-2                            DDYLDD----ERSLDKEQFALRKMHYVPS-------HYTDVRRRVLVGPKDSRPPLNDKV 1399
```

```
DQ286292 Rosy apple aphid virus                       SKRQYTSSNTSSSSSSSNCSESSTDSSDSSNLSHPSEHSIKITDNSPHTKHRGKIIPNNK 1118
OP265432 Penaeus vannamei solinvivirus                ------SSSE---------------SESDEEQSAPQK-SEK---------------SQTR 1384
MZ210029 Fushun monoleptalauta Solinvi-like virus 1  ------------------------------RVVKLRK-RAP---------------GTKR 1598
KX024775 Nylanderia fulva virus 1                     ----------------------------SDDHFDPRAWNAR---------------LAQL 1408
KY070327 Diabrotica virgifera virgifera virus 2      ------ESET---------------E-SEVENQKSLKGSKK---------------PSKK 1333
OQ540582 Apis mellifera solinvivirus-1                ----YRRQAD---------------SSDDEEEDQRRKGSKK---------------GNPK 1385
MN918666 Picornavirales [unknown host]                ----RREVNT---------------SSDEDDYYEKRKTSRK---------------AAPK 1117
MW251313 Icha Creek insect virus                      ----------------------------EKEAIGDRKR--R---------------PKKK 1238
MZ822083 Apis picorna-like virus-4                    --------------------------DSECDIEQDRGK--G---------------GRKR 1131
FJ528584 Solenopsis invicta virus-3                   --------------------------------VQPQRKGEK---------------KIKK 1103
MW314636 Myrmica rubra picorna-like virus             -------------------------YDEDGNVVDEAKK-NK---------------PRKK 1008
OL569508 Corparats virus-2                            NPSEYSDTDI---------------SDNEENIIHGRQLDQN---------------EINY 1429
```

```
DQ286292 Rosy apple aphid virus                       IEQIKQPDNDETQKEDYTKL-PHEHV-CTTCGCLFTHIHKFNKNF---LHPTYQNACPNY 1173
OP265432 Penaeus vannamei solinvivirus                ---VA---RKSKQSAPEKKERPNTKVL--------RKQKQSEPEHNLKSSTKVARKKPQS 1430
MZ210029 Fushun monoleptalauta Solinvi-like virus 1  ---VKKVTTDDDCKHQGKWD----------------DSSSDESDF--GIQKKRQPGNKRS 1637
KX024775 Nylanderia fulva virus 1                     ---QARYPNHSNEE---------LQAA-----------------------------M--- 1424
KY070327 Diabrotica virgifera virgifera virus 2      ---VG-QASDLDKKVPQ-------------------------------KKKLTGAKKPAK 1358
OQ540582 Apis mellifera solinvivirus-1                ---SKKYDSDDDKKRQC------LQEG---------------------RKKATKKGNPKT 1415
MN918666 Picornavirales [unknown host]                ---NRRFESDDDRRREF------LDSVYS-------------------ERKKSRKAMPKN 1149
MW251313 Icha Creek insect virus                      ---VIHSDTEEEEAVGDRKRKPKRKVIHSG--------SEDEEAV--GDRK---KKRGTK 1282
MZ822083 Apis picorna-like virus-4                    ---NPKYYSDVEQDKGKGSRRKNPKV-YSD-----IEQDNF---------------RSRI 1167
FJ528584 Solenopsis invicta virus-3                   ---VTNYDSDGVQPQSNVKVEEEIKLVFDPTGQKLLFGNDFTSEL--ETLVELEKDDEEF 1158
MW314636 Myrmica rubra picorna-like virus             ---IVKYDTDEDERQAPRKR---------P---VKK-------------------VNPTW 1034
OL569508 Corparats virus-2                            ---ILGYKHDL---KKGKKR---------P---VLKDGNDTEPER--RSHKK-GKKRPIL 1468
```

```
                                                              .

DQ286292 Rosy apple aphid virus                      KCS-NYHSGHNKYSAKLIQVDFISSANEPLTPNAMTKLMGEEYAPVRVSLLDFFSTLEPE 1232
OP265432 Penaeus vannamei solinvivirus               EP---QKS--DKS------------------ST------------------KVARKLMQT 1449
MZ210029 Fushun monoleptalauta Solinvi-like virus 1  PRDYSTDFDNRKN------------------EKEKKRTPGQPK------TVKDYDTDYEN 1673
KX024775 Nylanderia fulva virus 1                    -----A-QADEKG---------------------MRV----ET------CLLWQKKLNPE 1447
KY070327 Diabrotica virgifera virgifera virus 2      KT---GDIS-----------------------------------------------DLDK 1368
OQ540582 Apis mellifera solinvivirus-1               KK---YDSDDD-----------------------------------------RTP---QN 1428
MN918666 Picornavirales [unknown host]               RR---FESDDD-----------------------------------------RKREITQN 1165
MW251313 Icha Creek insect virus                     ----VVHSDDD-------------------------S--------------PVRQLKVIG 1299
MZ822083 Apis picorna-like virus-4                   STEEVSRAE-VKQ---------------------LAS---------------LIDCKRQG 1190
FJ528584 Solenopsis invicta virus-3                  T---KSKID-NKS---------------------MAGLRREVR------RRRYARSKKAQ 1187
MW314636 Myrmica rubra picorna-like virus            SD-----ADNGKQ---------------------MKQ-----L-------LDEFLTRQGS 1056
OL569508 Corparats virus-2                           KNGNDTDIEKEKH---------------------ISGPL--SL------TQRYQLKLQSS 1499


DQ286292 Rosy apple aphid virus                      ERKRVYDYINQARDANDKTFYRLCKRYPGLENCIMKVVHANMLTDADMI----VNDDTDL 1288
OP265432 Penaeus vannamei solinvivirus               ---ILEEDEEVPQSLYDFEVT-----------------SRNPES-VDTTI-------RLL 1481
MZ210029 Fushun monoleptalauta Solinvi-like virus 1  ---RKKQSVLALPDMNAWSMFD-----STQD-VISR-SHKNLAM-LCMFPKGMYEADSIL 1722
KX024775 Nylanderia fulva virus 1                    ---LQDQSVNNQEPMSRFDNTR-----LSNNVIYPV-IHRNLCH-VYMSM-------SDL 1490
KY070327 Diabrotica virgifera virgifera virus 2      ---KQKQTVNKMTDMNSYDAFK-----AHIDACRKQ-ALKAMVQ-IYVLP-------DDG 1411
OQ540582 Apis mellifera solinvivirus-1               ---CQNEDVKTLCDMNDFTPYK-----LYMQNAYLR-ARTNMLQ-VYTVY-------DDR 1471
MN918666 Picornavirales [unknown host]               ---STKEDVLKLRDMNDHTSFL-----PLMENAFKR-ARKNLCQ-MYCSD-------STA 1208
MW251313 Icha Creek insect virus                     ---REREDVLTLTDMNGFDSYV-----PIIKNSWTK-TRKNMTM-VYMSN-------AKD 1342
MZ822083 Apis picorna-like virus-4                   ---LEKQDVRTLCDMNDYDAYK-----KYIENAYTK-ATKNLAQ-VYMVA-------SDV 1233
FJ528584 Solenopsis invicta virus-3                  ---IEKQEVLTLPDVNGFEGGK-----PYFQIAEEK-ARKNLCQ-IYMIA-------NNE 1230
MW314636 Myrmica rubra picorna-like virus            ---IQRQDVKTLVDMNSYDAYK-----PYIDVAVTK-ARKNMTQ-LYFVH-------SQE 1099
OL569508 Corparats virus-2                           ---KRKQYIKDLENMNNYDSYM-----PYCENAHNK-VTKNLVQ-VYIND-------AKE 1542
                                                           :

DQ286292 Rosy apple aphid virus                      EILHKKLNKAYVQISYAGFRAYALHVGEGKFLSVAHCFDGVGK----SLVLLSD---GK- 1340
OP265432 Penaeus vannamei solinvivirus               AENYVKV--------KGFGSNFGIILGNGFILTTLHTVRMNTI--DSLIEIDSDTLF--- 1528
MZ210029 Fushun monoleptalauta Solinvi-like virus 1  KINPSTV--------SGFLSVYALFVAENYFVTVNHALQN-----NYNIFVA-EDFGVAD 1768
KX024775 Nylanderia fulva virus 1                    GDTTQED---------LKAVNYGIFVKSRTLITVAHSFKDHKD---WYWYVGCDELGEK- 1537
KY070327 Diabrotica virgifera virgifera virus 2      EILHYEP--------RGNQSCYGLIVGDDMLVTVGHVVDSVEERPGSSVYACCDEFEEEA 1463
OQ540582 Apis mellifera solinvivirus-1               DIIDFEP--------IGKQSCYGLFLTGTMFATVGHSVPA-----AGNLYACSDSFDKA- 1517
MN918666 Picornavirales [unknown host]               QILQREP---------DYNCCYGLFLGKNMLVTVGHLVDV-----DMGVYVGHDDLEGF- 1253
MW251313 Icha Creek insect virus                     DILTKEP--------AGKQACYGIMINDKLMVTVGHTYETWKQ-SGMSLHMGADELNNK- 1392
MZ822083 Apis picorna-like virus-4                   PVMNVEP--------IGQQMCYALFLRGNMFVTVGHIADNISG--WFHLYVGCDSFGGK- 1282
FJ528584 Solenopsis invicta virus-3                  NCIASKF--------SDHIVCYGLFVFKKRLASVGHIVEALKCAPGYNLYAGCDQFNGK- 1281
MW314636 Myrmica rubra picorna-like virus            SKLYSEP--------IGEQACYGLFIRKNLLVTVGHVAKDIKRMPAYNLYVGCDSFNGV- 1150
OL569508 Corparats virus-2                           DIINYEP--------KDGLANYAVFIKKNLLVTVGHLVDQMKLYIGCRLYVGCDQFKGK- 1593
                                                                          :.: :    : :. *                 :

DQ286292 Rosy apple aphid virus                      ----NYPGVVVSITR--ERDLSIIYCKELS--NL--KSARSAFVKDS--SDL-------- 1380
OP265432 Penaeus vannamei solinvivirus               ----SAPATIHTTWE--GSDLALLKCPKATG-----KALLKHWVTNDDIHKLYEGAMISP 1577
MZ210029 Fushun monoleptalauta Solinvi-like virus 1  NVITLYPVKVHQRNA--LKDVAFCSCT--MPKSF--KNITSNFISRKHIEDLA------- 1815
KX024775 Nylanderia fulva virus 1                    ----KYKVKLTNKFVGTNRDLAMCEIISSKAPQF--SDITKHFPSRAEMGRW-------- 1583
KY070327 Diabrotica virgifera virgifera virus 2      GSFKSVRLSLIHRYP--SRDISVWYLNPKL-ANYKPQSLRKRFVYRKKIYDE-------- 1512
OQ540582 Apis mellifera solinvivirus-1               -----HKCHLLYKYE--YRDLSIWYIPSLE-KKFCAKSIRKHFLSQDYYEDD-------- 1561
MN918666 Picornavirales [unknown host]               -----HRCTLLLKYP--DRDISVYYVPSVR-YAF--PDISKHFLPRHVYSEQ-------- 1295
MW251313 Icha Creek insect virus                     ----FYKMRPVQHYK--HRDISVWTPIPKESLPF--KSIANLFKPRKEVVQW-------- 1436
MZ822083 Apis picorna-like virus-4                   ----FYRCKLLKTYK--SRDLSFWSCD--IKQSF--PDITKLFLTRKQMYDY-------- 1324
FJ528584 Solenopsis invicta virus-3                  ----LYKMNLVRNYR--KRELSVWDVD--CPNDF--VDLTSFFIPKEELYDA-------- 1323
MW314636 Myrmica rubra picorna-like virus            ----FYKAVCRNVFK--ARDLSAWTVE-GIP-DF--PDISNLFVKKKEIYEMT------- 1193
OL569508 Corparats virus-2                           ----FYKARLLRNYK--LRDLSVWEVE-RCPFDF--PDISNLFFSRDEIRDG-------- 1636
                                                                        :::                  . :

DQ286292 Rosy apple aphid virus                      ----TLGGYFMR---CGPSVTIVAGNIEYREYQEFIDGSCSNEYYRPSM----------- 1422
OP265432 Penaeus vannamei solinvivirus               NSSRGVDTFNGRFILFPQ-----------------------------KR---RLGQ--YH 1603
MZ210029 Fushun monoleptalauta Solinvi-like virus 1  --SRKHETAFAR---YAK-----------NKQLETSLGVGE--FFIRKTDISYFDGSVEE 1857
KX024775 Nylanderia fulva virus 1                    --T-TRSFHVQFFRYFGVPEAL-------NKVTVQVQAHVG--KMTAHFNTTSVKTSDKV 1631
KY070327 Diabrotica virgifera virgifera virus 2      --T-VISAVLER---FGT-----------NKQREYHCGDLN--FFTGPD---ACGKG--T 1548
OQ540582 Apis mellifera solinvivirus-1               --I-TANAVIQR---FGP-----------NKTEEFHSGECT--MFRTPF---YFGKNSKK 1599
MN918666 Picornavirales [unknown host]               --N-VWDSVIQR---FAP-----------GKKEEFQSSTFE--YVCGPT---PFSGGSTT 1333
MW251313 Icha Creek insect virus                     --H-ELSAFFQR---FGS-----------NKKEEFARGPLA--IQTDPT---SDGFG-GV 1473
MZ822083 Apis picorna-like virus-4                   --Q-MVNAVIQR---YAP-----------NKEKIWIQGCCD--IVQPYY---STGS--GG 1360
FJ528584 Solenopsis invicta virus-3                  --E-NCNTVLGR---FGM-----------NKREVYLYGNCE--FIQEFF---KVDN--KG 1359
MW314636 Myrmica rubra picorna-like virus            --E-GVNTALQR---FGP-----------NKEEIWVQGSAE--MISEYY---QLDK--DG 1229
OL569508 Corparats virus-2                           --Y-MINSVICR---YGP-----------NKKEIWLLGMEE--FVKPFM---PLTE--GG 1672

                                                                 Protease domain
DQ286292 Rosy apple aphid virus                      -EVLKLRRVGLVVTETIKKGDCGFPFVARIHG---TMRIIGIHNAYKTHT---VVFGSFI 1475
OP265432 Penaeus vannamei solinvivirus               DERTVSIDFVRSD-IVTRAGDCGSAYISTQKD--VRGHIFALHACLQNGR----SHGAYI 1656
MZ210029 Fushun monoleptalauta Solinvi-like virus 1  AKDFAEITFGNSKTPRSTAGDCGLPHYLVTKGLDYSNKILGIHFAGTTGHGTSQMNSSLI 1917
KX024775 Nylanderia fulva virus 1                    KADYA-MAVMEGNHAYTSFGDCGLPYFAISSQ--FQNKCVGIHCMGSVQGTPSKGMASMV 1688
KY070327 Diabrotica virgifera virgifera virus 2      LSDYGQIEYGTFGIKLTTYGDCGLPYYACEPQA-CEQKILGIHCMGNNEGYTSCGVVATI 1607
OQ540582 Apis mellifera solinvivirus-1               MGDYAQLSFGTSNIKLTTFGDCGLPYYFCETDPKYHDKIIGIHCMGNQLGYTSLGISCMI 1659
MN918666 Picornavirales [unknown host]               LRDWAGCYFASTGIKVTAGGDCGLPYYSIENHPILKDKIFGIHCMGNNEGQKTSAVAAVI 1393
MW251313 Icha Creek insect virus                     ISDFGEMSFGTFGTKATTYGDCGLPYFSAEPH--FNDFILGIHCMGNVEGNYTTSIAALL 1531
MZ822083 Apis picorna-like virus-4                   ISEFGYVDFATIGIQLTYYGDCGLPYYVAEKDF-AHDKILGLHCMGNVDGYSSVGIVALI 1419
FJ528584 Solenopsis invicta virus-3                  AQEFGYIDWATVDITLTTGGDCGLPYYICERKK-FHNKIMGLHFAGNNVNHKTIGMSALI 1418
MW314636 Myrmica rubra picorna-like virus            IQEFGIVDFATFDLMLTNAGDCGLPYYPAERTR-LHNRILGIHCMGNVTGYNSIGIMALI 1288
OL569508 Corparats virus-2                           IEQAGIMDFGIFGTPLTTYGDCGLPHLSIDKC--HQNKIIGIHCMGSTEAHPSIGVSSLI 1730
                                                                        ****  .             ..:*              . :
```

```
DQ286292 Rosy apple aphid virus                      ------------SQ--------------------------PLIDELCSVVVPNADV---- 1493
OP265432 Penaeus vannamei solinvivirus               GREDLQDAMQSTPR------------------------RIPQSCPTPAEGV--------- 1683
MZ210029 Fushun monoleptalauta Solinvi-like virus 1  FKEELEEWIEGLSKKK--------------------NSNEPVECPGCKVVKSGDLVSLPL 1957
KX024775 Nylanderia fulva virus 1                    FLEDFEGLKNQSWG-----------D--------IGVTEDSDTCNICDTNTS-------- 1721
KY070327 Diabrotica virgifera virgifera virus 2      FEEDYAVWLKQKET-------------------FLEKAQQSQCCKMA-TKKWQLLP---- 1643
OQ540582 Apis mellifera solinvivirus-1               FKEDVVDWEALYQKVRSQQVSQSWNNPIVRAEADGEGPGKSPCCEFD-VKTITIKA---- 1714
MN918666 Picornavirales [unknown host]               TKEDITDWQRRLGQ-------------------KEIIMGENACCKFH-VDRPTEKP---- 1429
MW251313 Icha Creek insect virus                     YEEDIRAWSKINLK----------------------E-ERKVCCDFAADDTYIQIP---- 1564
MZ822083 Apis picorna-like virus-4                   MQEDFADLD---R---------------------------QTACDFCSVTKTRLAP---- 1445
FJ528584 Solenopsis invicta virus-3                  YKEDLVVWKGAERQ---------------------------SKCKFCDVKDIIIAQPDIP 1451
MW314636 Myrmica rubra picorna-like virus            YLEDIQLWSQFCKQDV-------------------------ISCRHCGETNVMISP---- 1319
OL569508 Corparats virus-2                           WKEDIVEWMGDISKPL-----------------NKQG-SLQEPCDFCQVDLPVIKRAKVS 1772

DQ286292 Rosy apple aphid virus                      ---PS--TAVVV------------------KVPIVRDYTG----------SGYVEMSMPM 1520
OP265432 Penaeus vannamei solinvivirus               -------SHI---------------------------------EINGHEMLGTT--SQVD 1701
MZ210029 Fushun monoleptalauta Solinvi-like virus 1  PEKEVCENHTIRWDEDHDNSFTCFSRHLTFLK---DTFSGSCEMNHGEHVPHSVHHPHTH 2014
KX024775 Nylanderia fulva virus 1                    --VTQDCGNIIIWDSLHTFPKMSWQNHVRSYLRMFNSTHAVVFTYNWGTLWGSVKHQHTK 1779
KY070327 Diabrotica virgifera virgifera virus 2      DTTPKGDGHVTLWNPTHESTRGSFANEFAYLITTLQNKQGRYIKNSG-KLHGSIEHSHLQ 1702
OQ540582 Apis mellifera solinvivirus-1               DDKPKCEGHETIWHHMHLSTISTFVDEVMTFANQRKQLRGVIIKNSG-KLYGTVEHSHTQ 1773
MN918666 Picornavirales [unknown host]               DDHPKAKGHVNVWHSEHTSTWMTFSEEIKEYLTKHPRYEGGILKNSG-IMYGTIEHSHTQ 1488
MW251313 Icha Creek insect virus                     -TVKKCPDQFNVFSSQHNSSNRTFSEELAAGLEFQPGFNGVILKNSGQIAYGSVKHSHTQ 1623
MZ822083 Apis picorna-like virus-4                   ASIPKCDNHEIVWNGNHESNAKAFVEELKFHDELRTSFTGMVIKNSGPLLRGSVSHSHTQ 1505
FJ528584 Solenopsis invicta virus-3                  KEKYKGYNHEIVWNSLHESSPTTLNEELEHYLNIFPKFTGTIIKHSGDKFYGSVKHSHTQ 1511
MW314636 Myrmica rubra picorna-like virus            SLTPKCDTHEIAWSPVHESSPATFSEELNGYFKLRGNFTGQIIKNSGPRFYGSVEHSHTQ 1379
OL569508 Corparats virus-2                           KSQDKNNKHEVLFGDVHDSSPATFYREIKQFLDVNKTFTGTVLKNTGNKLGGSVEHSHTQ 1832

DQ286292 Rosy apple aphid virus                      EYAQEFKKC----VTPDPYMPR-----------------------NSGL----------- 1542
OP265432 Penaeus vannamei solinvivirus               FLVQKFGST------DAPRMPL--PKDAIELGRLAKTPPETTKSRKIPITQPGMQTT--- 1750
MZ210029 Fushun monoleptalauta Solinvi-like virus 1  CMPFYFKPEYEKYAQNSSRWVK-HPTGEYSYWIYA----------QTTATISAIQNKYLK 2063
KX024775 Nylanderia fulva virus 1                    FYADHTRWM----DDTKEGV---YPAHEVGLDHRTKD--EITVVKFENVSMATLQEFLR- 1829
KY070327 Diabrotica virgifera virgifera virus 2      FIPNTYHDL----EV-PHDWLL-TTAGELEIANGME-SSQKLSMKTAITGLENFADHILR 1755
OQ540582 Apis mellifera solinvivirus-1               FLPNQRLEV----TS-PHGWLI-KSTTDMGIDRLP---NTKVFVRTSALPFASFASHFSR 1824
MN918666 Picornavirales [unknown host]               FLPEQDPTS----NHDVSPWKV-VDAKDVWDRGTG---QCELITVEIGGDSSSLTKYLKK 1540
MW251313 Icha Creek insect virus                     FLPDETI-S----FNKEPGFKGSMTFGEYGEDSSHIPPNTKVVVTRKNIELVNMPSFIK- 1677
MZ822083 Apis picorna-like virus-4                   FLSGMRDET----FVVTRGWVT-TTASELGFDSPRINPNSTFSYRVIDVSYGGLYTCLN- 1559
FJ528584 Solenopsis invicta virus-3                  FIS-KFKTE----LTVTNGWKL-STAGDCQFESNHISPNTEVMYRVVDVQFNSIFKAFK- 1564
MW314636 Myrmica rubra picorna-like virus            FLHNVFRDN----VKVTNGWRT-STAEDLDMHHGRIDAGETISYCVKDVPFPAIYNMFN- 1433
OL569508 Corparats virus-2                           FIFNEFE-N----LKISNGWKT-ATASEIGFNKLHVSPKETVSYVVKDCQIESIYSTFN- 1885

DQ286292 Rosy apple aphid virus                      ---TSIGFNLKFKNYN---RMKEKH--------------YT----------HELQLE--- 1569
OP265432 Penaeus vannamei solinvivirus               ------------------------------------------------------------ 1750
MZ210029 Fushun monoleptalauta Solinvi-like virus 1  LHSTKQILRSSPTRNKKTGEIQKMRREYFLIAYPDSKGNYG-------------MELKEP 2110
KX024775 Nylanderia fulva virus 1                    -NDTIIGFRFDGFV-----RLRNDNILITTDIYVHYETNFTNQSCDI------------- 1870
KY070327 Diabrotica virgifera virgifera virus 2      NKYWGDDFRCNFNVYT---DIKSGLPMVKYCIITTH--------------INKFDKSPQE 1798
OQ540582 Apis mellifera solinvivirus-1               LTDLGNDFRIKGCIYN---EPVTKRLMFRFTLIVLFDSVSQ-KRCEIITALNTIQCINES 1880
MN918666 Picornavirales [unknown host]               YWERSSDFRIRAYRYK---HFKTNAFVIKMTIIVPYDKDYK-Q--EIFAD---FIEETQP 1591
MW251313 Icha Creek insect virus                     -KIKTSNFRLRINCLS---GEKMMKFEIEL-IEFPV---------------TKLESYK-- 1715
MZ822083 Apis picorna-like virus-4                   NIGYASNFRLYANVYN---KG-HKRAALIK-IIIPMEEVPS--------RESQFSLSK-- 1604
FJ528584 Solenopsis invicta virus-3                  SQPYIKNFRLIANVYE---KDGKQRVTILT-IIPVSDFNVK------------QQTVR-- 1606
MW314636 Myrmica rubra picorna-like virus            AISFAKNFRLNIDVFI---NKENKRRAIIS-LFVPADEAQK-K--QIIEYGPLLNFNR-- 1484
OL569508 Corparats virus-2                           VVPHVKDFRIRINIYI---TESLKRRATIG-LYILHNSKLP-Q--------TELDLTK-- 1930

DQ286292 Rosy apple aphid virus                      ---------------------------------------------------NE------- 1571
OP265432 Penaeus vannamei solinvivirus               --------------CSKVPVSTDLYRVLE------------------------------- 1765
MZ210029 Fushun monoleptalauta Solinvi-like virus 1  QIGMKDRTLYVKQQKVVVPPCVENIEILEEPIQ-NIDNGIPWDIPFQE---GGKVSMIGS 2166
KX024775 Nylanderia fulva virus 1                    ------FSKVNLPSGDSGYILTELMPIYRGAQI-NFLNGKLNDTPFDQIKDNETVKVFGT 1923
KY070327 Diabrotica virgifera virgifera virus 2      QIY--MSALPELPFGNQVYASEEVKDIITTSLM-EKQRGVSEDMPYKLVSSNETVRVIGV 1855
OQ540582 Apis mellifera solinvivirus-1               N-E--DTFAIEPPFGGPVYVSQAVKEIIDTATL-TRKRGHPEDVPYENVKDNETVRVLGS 1936
MN918666 Picornavirales [unknown host]               N-D--TYVPFTSNFGAPVYVPLEVNQVLQATIR-NRERGKLEDVPFARVPGNETVRVLGA 1647
MW251313 Icha Creek insect virus                     --R--EVVFHQFNDKKEAYVCEELKEILVEAKANLENQGKLPDIPFVQDENNDTINVIGV 1771
MZ822083 Apis picorna-like virus-4                   --Q--AIVPLNINDEQVVHVNGDIFDVMNQHMQ-RLRRGMLPDIAYEDIPDNETVQVIGV 1659
FJ528584 Solenopsis invicta virus-3                  --Q--ALVPLHLNEDEEVYVTEDVSDIFKTAIK-RKQRGILPDVPYETVEN-ETVEILGI 1660
MW314636 Myrmica rubra picorna-like virus            --Q--GNIPLPISETEEVYVSGDISDIANQYIA-RKRRGFLPDIPFEKIENNNTVSVIGI 1539
OL569508 Corparats virus-2                           --Q--GMIPLGLTDDQETYACDDIKTITGIYQK-RYQRGEFPDIPYDKMEDNSNVQIVGI 1985

DQ286292 Rosy apple aphid virus                      ----------N---------------------TCLPAAFSNRY--VTDTSELVENGYGQP 1598
OP265432 Penaeus vannamei solinvivirus               ------------------------------------------------FDQMAKELNGKP 1777
MZ210029 Fushun monoleptalauta Solinvi-like virus 1  FRYNQSHTPKNDYKRIGLST----NLPI----EKVPFCDDSEKAPPDQYEKLITDNFGKK 2218
KX024775 Nylanderia fulva virus 1                    FNRTFSHVPKSPYFQTPWFDLLKDSDGKPLLPTKLPMENDITLLTDEQIASLSTDRDGNP 1983
KY070327 Diabrotica virgifera virgifera virus 2      FRGDKSHKPTNTFKMTPFAKHIDKAFKL---EHKKPFSTDMKNIPEADLEKIVMDLNGEK 1912
OQ540582 Apis mellifera solinvivirus-1               FHQSQSHTPKDHYYLSPFSSIVKDLIPI----EKRPCIYELGKIPQDIQDTLARNNYDLP 1992
MN918666 Picornavirales [unknown host]               FSQSQSHVPNNHYQLSPFSPIVKDIIPI----EKKPCVYDMSLVPDQEKEVMVKNNYGEP 1703
MW251313 Icha Creek insect virus                     LPYNASRPPGDQYSLVPYSSELSKIIPI----TKVPFNKNSELAPAEQKEKMALNTWGLL 1827
MZ822083 Apis picorna-like virus-4                   AHRNMSPLPPNSYMPTPFHMAVGDTLGA----YKLPVRFDANNAPQEVKDRMFCDRCGNP 1715
FJ528584 Solenopsis invicta virus-3                  THRNMTPEPAQMYKPTPFYKLALKFNLD----HKLPVNFNMKDCPQEQKDMMVLDRLGQP 1716
MW314636 Myrmica rubra picorna-like virus            AHRNMSPQPNAVYKVTPFINRLQN-IQP----YKVPVRFDSNNAPQEVRDTLALDRAGFP 1594
OL569508 Corparats virus-2                           AHRNMSPLPDGKFYPTPFSLRLEDETSP----HKYPARFDGENAPESEKQKMDCDLFGRP 2041
                                                                                                        :  :  .
```

```
DQ286292 Rosy apple aphid virus                      DPLWTQACKYGRRNKNYDKHIFRHAVEMAL-------DYNRMTYCADKPFKFLTEFEALN 1651
OP265432 Penaeus vannamei solinvivirus               SHLATQIAKHG---MTPDLSIDEGCLRRATSLVAYRYVSAMAKNG--RQLSTLSLQQTFG 1832
MZ210029 Fushun monoleptalauta Solinvi-like virus 1  SARATQSIQWDGDFRDVPAALR--------THVTEQVAYGMHEVY--AGLRVLSDHEVLH 2268
KX024775 Nylanderia fulva virus 1                    SARVTQALQWAHLLHEPDLNFF--------DEVSQEFMKYVNLIY--GKMSLLTDEQVLQ 2033
KY070327 Diabrotica virgifera virgifera virus 2      SQLATQSIQWAHRNHNPGNLFL--------KEITNEFQSKVLQYY--SNMDEMTDQEVLE 1962
OQ540582 Apis mellifera solinvivirus-1               CQRTTQALQWAHPNYVIDEELG--------KVIDDEFSQKILRYY--SGLRLLSDEEVLG 2042
MN918666 Picornavirales [unknown host]               CQRTTLSVAWAHKICGER-HFI--------AEVGREFQKKIMQYY--SGLTLLDDRQVLE 1752
MW251313 Icha Creek insect virus                     CPRTTQALQWAHKHYSPASDIR--------NYVKEQMYSKAKAYY--ADMKVCNDEFVFK 1877
MZ822083 Apis picorna-like virus-4                   DPRITQSIAWAHPNFSPDIELR--------KFVKSEYMANILESY--SGMNLLTDHQLFH 1765
FJ528584 Solenopsis invicta virus-3                  NPRITQSLKWAHKDYSPDYELR--------KYVKEQYMCNIMEYY--AGCNLLTEEQILK 1766
MW314636 Myrmica rubra picorna-like virus            SARITAALKWAHPIISPPIDLR--------KYVLNQFRSNILEYY--ANLNLLTDKQVLS 1644
OL569508 Corparats virus-2                           DALITQARRWAHRIVAPNPYLR--------SFAKSEYRSNILAHY--SNMSLLTDHQVLH 2091
                                                         *               :                                     :

DQ286292 Rosy apple aphid virus                      GRTE-------PFLSNVDISTSAGPYAKYFHNIYTKREILDTELADGKPIYRFSQNRAGQ 1704
OP265432 Penaeus vannamei solinvivirus               GIKA----KGKVTLDKLTNDTSAGFLSLYLNKAPLKDAYVKV---HDDGQVE-----PTE 1880
MZ210029 Fushun monoleptalauta Solinvi-like virus 1  GTGD-------GGLERLNLDKSIGWTMSSLYEVKKKSDVLSV---DNLGKVSWVDTPAGR 2318
KX024775 Nylanderia fulva virus 1                    GFPKKH--PYVDCLGSLQLNASIGWSMKALFNVNKKNDIFTK---DEDGLITLNNNDAAK 2088
KY070327 Diabrotica virgifera virgifera virus 2      GCSLTARPKDAPYFCGMALDKSVGFTMKELFYVQKKSDILTV---DELGHYKWLENEAAH 2019
OQ540582 Apis mellifera solinvivirus-1               GLPK--SDPDHDYFKGMELDSSIGFTMKNLYKVMKKSDVIHC---DENGKYTWLNNPASA 2097
MN918666 Picornavirales [unknown host]               GIRY--PDPDAQFFKGMELNTSIGFTMRQLFYVTKKSDVIGC---KEDGTYFWQHNAAAD 1807
MW251313 Icha Creek insect virus                     GYPITH--RFREGMKGLELDSSIGFSLKSLFCVNRKSDVITC---NESGEYFWKDNEASL 1932
MZ822083 Apis picorna-like virus-4                   GYESGH--RLRGGLQPLEIDSSIGWTLKQIFNVTHKSDVIHL---SDKGVYSWKDNECAD 1820
FJ528584 Solenopsis invicta virus-3                  GYGPNH--RLYGALGGMEIDSSIGWTMKELYRVTKKSDVINL---DSNGNYSFLNNEAAQ 1821
MW314636 Myrmica rubra picorna-like virus            GYENKH--RLRNGLQGMELDSSIGFTMKQLYTVQKKSDVIDL---SATGEYSWADNEASE 1699
OL569508 Corparats virus-2                           GYQSTH--SLGRGLDGLTLDSACGWTLKQLYTVNKKSDLISV---DMKGNYAWEDNEAAQ 2146
                                                     *            :  :  . : *     :     *   .
```

RNA-dependent RNA polymerase (RdRp) from the positive-sense single-stranded RNA [(+)ssRNA] AmSV1: 2103-2418

```
DQ286292 Rosy apple aphid virus                      CIRNHLKTQKNLLATYGIPPCLISQDN-----AKVENIEKEKAQKGKVRLFNNVDPAVNA 1759
OP265432 Penaeus vannamei solinvivirus               LGIKTFQDANNILA-RGKDYPNATLLSIGHCKLKSELLPAQKIEQGQLRCFVAEGIESII 1939
MZ210029 Fushun monoleptalauta Solinvi-like virus 1  FAKQFYQEGVELIK-NGIRPWSVFLEM-----MKMEKLKLAKQY--IGRIFCSEDIHGVL 2370
KX024775 Nylanderia fulva virus 1                    AFWEMFQKAKELVN-NGEPVLVTVEEC-----GKMEKLKVSKYH--IGRTFCSMDFLNIL 2140
KY070327 Diabrotica virgifera virgifera virus 2      WLREQFQLAKSVYA-RGEQYFIAFLEL-----LKMEKLKESKRF--VGRTFLAQDILGVL 2071
OQ540582 Apis mellifera solinvivirus-1               YLKQEYQLAKDLSD-QGKNYYVAYLEL-----LKMEKLKLSKIY--TGRTFVVQDTLGVL 2149
MN918666 Picornavirales [unknown host]               YLKQRFQEAKVLAS-EGKKYVSIFTEM-----LKMEKLKLTKIF--RGRTFVAQDVLGVL 1859
MW251313 Icha Creek insect virus                     YTQNLFKEYKELSD-QGKDYTVCFNEL-----LKMEKLPPHKNF--LGRTFQVQDLIGVM 1984
MZ822083 Apis picorna-like virus-4                   FAKSMFEVSMMQAE-AGIRYWTVFNEL-----LKMEKLKPEKQF--LPRTFTANDLNGVL 1872
FJ528584 Solenopsis invicta virus-3                  YTQELLKISMEQAH-NGQRYYTAFNEL-----MKMEKLKPSKNF--IPRTFTAQDLNGVL 1873
MW314636 Myrmica rubra picorna-like virus            FLQEMYHDSKQLVS-QGKRPWIAFNEL-----LKMEKLKPEKQF--LPRTFTAQDLLGVL 1751
OL569508 Corparats virus-2                           YLKYMYYQAKELAK-DGKRYWSVFNEL-----LKAEKLKKEKMW--IPRSFIVEDFLGIL 2198
                                                                     *                * * :   *      * *   .

DQ286292 Rosy apple aphid virus                      ILKIMFGDWFSRAMAKSSDGYYAIGQNPYTTSTEIWHRFSTKQGKILNTDFKAFDKLLIT 1819
OP265432 Penaeus vannamei solinvivirus               PCRSKLGSFVAMQNELRHDLFPCIGIDFETESSYLLHRLLSVNDTIQQGDFKRFDKTMPI 1999
MZ210029 Fushun monoleptalauta Solinvi-like virus 1  WGRKFMGDFVRRA--TLYDDGIGVGVDSNADFDRIYHK-LVEKGLYWTGDYSRWDKTVPL 2427
KX024775 Nylanderia fulva virus 1                    LERYVMGYFSAKA--MRDDDYCAVGIDPYANFHDMFVE-LRKFDHVFAIDYKRFDKTIPQ 2197
KY070327 Diabrotica virgifera virgifera virus 2      LERKYMGEFAIRA--MRDDPNCGVGVDAHKDFNRVYNY-LNHFQFTWAADYKRFDRTIPG 2128
OQ540582 Apis mellifera solinvivirus-1               MERRILGMIAVRA--MKDDITCGVGTDPFKNFHQYYLQ-LSQFQNIWAGDYKRFDRTEPA 2206
MN918666 Picornavirales [unknown host]               MERRILGGMCIAA--YKKDPNCGIGCDPAVDFNRFFIR-MNKFSNLWAGDYKNFDRNTPG 1916
MW251313 Icha Creek insect virus                     MERRNLGEFSARA--MILDPTCGVGTNAYSDFHKIYLR-LRKHPNLFTGDYKRFDKTVPM 2041
MZ822083 Apis picorna-like virus-4                   MERKILGEFTARA--MLWDKTCSVGINVYSDFDQLTRE-MSKFKNQFTIDYSKYDRSIPA 1929
FJ528584 Solenopsis invicta virus-3                  MERWILGEFTARA--LAWDENCAVGCNPYATFHKFATK-FFKFKNFFSCDYKNFDRTIPK 1930
MW314636 Myrmica rubra picorna-like virus            LERWVLGEFTARA--MLWDKNCGVGINPYTEFDKLFRY-LSQHPNVFTGDYKNFDRLIPV 1808
OL569508 Corparats virus-2                           MQRYILGEFCARA--KLNDPNVGIGINPYTQFDKHANY-LMKHPNMFTGDYKNFDRTIPL 2255
                                                       :  :*           *    :* :                      *:. :*:

DQ286292 Rosy apple aphid virus                      ELIEAFCYIG-GSLTKNEKNPHSELMYEAISLTLIHAVHILNGSVYVVNNGNESGTFVTT 1878
OP265432 Penaeus vannamei solinvivirus               ELKRAACEVVEKCYMRNPLNRYDAPARRVLYNTWCSPLYLARDLLMTTGNGQPSGNAMTA 2059
MZ210029 Fushun monoleptalauta Solinvi-like virus 1  WLYRIACDVLIKL------NPHLEKEIRTIVDMSSKTIHVSGRSMFYVSGGMPSGSFWTA 2481
KX024775 Nylanderia fulva virus 1                    FLIDLVFDCLIGV------NKKMEKPLKSMKKSFRHRIQISGNSLFETIGGMPSGSFITA 2251
KY070327 Diabrotica virgifera virgifera virus 2      CVFQQVRDMLVKH------NPKIGKQIYSVFNSLIYRFQITGRTLSQVYGGMPSGCTLTA 2182
OQ540582 Apis mellifera solinvivirus-1               DVFLRISKLLQKA------NPHMAKQIKSAFNAIVYRFQVSGTTLAHVQGGMPSGCTLTA 2260
MN918666 Picornavirales [unknown host]               LVMLEVMQCLKRA------NPHMANDIDSCMRTNIERLQVSGTTFFEVFGGLPSGCFSTA 1970
MW251313 Icha Creek insect virus                     VAFEDMIKILVRV------NPYIQNQITSTIKAIYNRIYVSGSTVATAVGGMPSGCVITA 2095
MZ822083 Apis picorna-like virus-4                   CVFEDICELLIGA------NPHLKHHLNSCFKTLVNRVQVSGNTIALVKGGMPSGCIPTA 1983
FJ528584 Solenopsis invicta virus-3                  CVFEDFRDMLIQA------NPHMKNEIYACFQTIIDRIQVSGNSILLVHGGMPSGCVPTA 1984
MW314636 Myrmica rubra picorna-like virus            SVFDDIRDLLIEA------NPHIKNEITSTFNSISKRIQISGTALTCTIGGMPSGCVVTA 1862
OL569508 Corparats virus-2                           CAFDDIRDLLMEA------NPHMANEIQVCIDCISRNFQIAANTLSYVVGGMPSGCLLTA 2309
                                                                        *                 . :    .  . .*  **   *:

DQ286292 Rosy apple aphid virus                      LLNCVSVHIIFNYSFIV-C-WNKVPDYIHIKPMLKDIMSRSELAILGDDKTQVVSKDIPM 1936
OP265432 Penaeus vannamei solinvivirus               TLNSIVSEIAYTYCLFRHA-------D---EHNLRLNTGDFTMIIYGDDWVAATKTELMP 2109
MZ210029 Fushun monoleptalauta Solinvi-like virus 1  PMNSFINRCLIYTVYCVLFAEKFG--C---FPDWQDFITNVYVMAYGDDIVVAVTPE-RN 2535
KX024775 Nylanderia fulva virus 1                    PLNSVFNLLITFAAFVYIL-KLHG--I---DATWEDFQRLVVCRFYGDDGVISVHESIAK 2305
KY070327 Diabrotica virgifera virgifera virus 2      PLNSLVNDYIIFSCFASIC-SDNG--M---EWNWQLYDKNVRRIFYGDDVYLSVSAKYSS 2236
OQ540582 Apis mellifera solinvivirus-1               PLNSIVNDYLLFSSYVALM-RKFN--L---AFSWEHYEANVKRLFYGDDVLVSVSDELSE 2314
MN918666 Picornavirales [unknown host]               AFNSLNNEFLMYACFYYLG-QKFN--L---DCSWEDYEANIERLYYGDDVLIAVSDAWRE 2024
MW251313 Icha Creek insect virus                     TGNCFINEYLIYTCFVLLG-NVAQ--F---DVSWKSYNRHVERVFYGDDVIVSCSDDVAK 2149
MZ822083 Apis picorna-like virus-4                   PLNSKCNDFIIYSIYVSLC-WDSNHEE---FAYYAVYNRKVVRRFYGDDGWISCDDDIAD 2039
FJ528584 Solenopsis invicta virus-3                  PLNSKVNDIMIYTAYVNIL-RRADRGD---ITSYRYYRDLVCRLFYGDDVIIAVDDSIAD 2040
MW314636 Myrmica rubra picorna-like virus            PLNCKLNHIMIFTAYISIC-WKNDRNE---LATYARFVKLVHIVFYGDDVIVTVSNEIKN 1918
OL569508 Corparats virus-2                           PLNSKLNDYMIFTAYISLC-WKHGRDE---IANYVQYERLVHRRFYGDDVQLSCSNDVAE 2365
                                                       *.                                          ***
```

```
DQ286292 Rosy apple aphid virus                     ---EEEDLIDIAASLGMECTKAK--G---GLDDGKQINFCSRVLVWDEV-EQ-IVYPRLK 1986
OP265432 Penaeus vannamei solinvivirus              ---NADKLISYMSEIGLTLTSPIKTEPLKDFYPIEEMEFCSRTFHEHTS---LVVLARLK 2163
MZ210029 Fushun monoleptalauta Solinvi-like virus 1 FFTQE-AAARIINRLGLVMSPADKDGNLVDFEPMNKLSWISRYFRQLGN-YP-VIVGALK 2592
KX024775 Nylanderia fulva virus 1                   FFNRVTLAKAVAHLFGMNMSSANKDDELKPFDTWEEVNFISRYFRFLNG-RS-VVLGALK 2363
KY070327 Diabrotica virgifera virgifera virus 2     VFTRTFVASKLLELYGMVIDSSVKDGGVSDFDTWETGTWISRYFRKLDR-YPHFVVGALK 2295
OQ540582 Apis mellifera solinvivirus-1              IFTRDKVSIVMMELFGMVLDSAAKDGSVATFDNWETASWISRSFRKIDQ-FP-FIVGALK 2372
MN918666 Picornavirales [unknown host]              YFTRDEISSILLTQFGMVLDSAAKDGTSAKFDTWESASWISRFFRKLGR-FP-ITCGALK 2082
MW251313 Icha Creek insect virus                    FFDRITLSQQMLETFGMVLDSSQKDGTVVRFETFEQASWISRYFRKLKH-YP-FFVGALK 2207
MZ822083 Apis picorna-like virus-4                  FFNSVTVARKAKQLFGMTITPSSKQGIQLPFDEPDQISFISRFPRKLDR-YN-FIVGALK 2097
FJ528584 Solenopsis invicta virus-3                 IFNCQTLSEEMKILFGMNMTDGSKSDIIPKFETIETLSFISRFFRPLKHQEN-FIVGALK 2099
MW314636 Myrmica rubra picorna-like virus           FFNLVSLSLELKTLFGMIMTSSDKSGELKYFESWNEASWISRYFRKLDN-YP-FYVGALK 1976
OL569508 Corparats virus-2                          FFNLVSLSDELKIQFGMEMTASSKEGEKKFFETYDEASWISRFIRKADK-FP-FYFGALK 2423
                                                                   *:             :   .   :**           .    **

DQ286292 Rosy apple aphid virus                     KSSIIGLLYWFASLDRNQVRDNLMIALFEASLHEREFYDSVLRDAMLVSLEFGIDIR--T 2044
OP265432 Penaeus vannamei solinvivirus              KSSIEALLHYCYSLTTEAVQDNIRTALQFAVSYDEEYYNLILHDAQILVAHYGGTLPQPY 2223
MZ210029 Fushun monoleptalauta Solinvi-like virus 1 KISINAWLHWTKHETAIQIGSQYTSALYEASLWEKDYFEQIYADVKLGIKEFPQVKN--Y 2650
KX024775 Nylanderia fulva virus 1                   KETILSYFHFTKSLEPQHLSSLLEKAAEEASIWGEEFYNYVEDLIRTCIDCCPPLRK--H 2421
KY070327 Diabrotica virgifera virgifera virus 2     KISINSHFYYVTSLDATHIASLLDTAMIEAALWDEEYYNTILECVRLALKHMPSLRA--H 2353
OQ540582 Apis mellifera solinvivirus-1              KISIGANFHYVTSTDLKHIGDLMTKALFEASFWDMEYYQTILTAIRRAISHKPALGK--H 2430
MN918666 Picornavirales [unknown host]              KISIEANFHYVTSIDYSHLGSLMTKAQYEASFWGKEYYLKIQSAIRRCIEKEPRLGR--F 2140
MW251313 Icha Creek insect virus                    KESIFAYFHYVSETTPSHIGDLLTTAQFESALWDDDTFEKVQEGIKFVLEKNPNILKHNY 2267
MZ822083 Apis picorna-like virus-4                  KISISTHFHWTIDCSPKQLGEQLTIAQLEASFWEESYFLNVQEQIRVAIRKVPEIRQ--Y 2155
FJ528584 Solenopsis invicta virus-3                 KISIQTHFYYATDDTPEHFGQVFKTIQEEAALWEEEYFNKIQSYIQEIIRKFPEISK--F 2157
MW314636 Myrmica rubra picorna-like virus           KISINSHFCYVSSLKPEHLGALFTVAQWEAALWEDEYFNNIQENIRIAIRYKPNIGL--Y 2034
OL569508 Corparats virus-2                          KITIHTQFHYLTSLSPQHMGDIYTTIQEEAALWDQEFFNKIQRCIHVAVSYNKDILN--H 2481
                                                    * :*   : :        .          :     . :  :

DQ286292 Rosy apple aphid virus                     VAFTNYNQSRKRLKSMLMNDFEYQQISALA------DRKTKESDYSDLIRILKTAQLLES 2098
OP265432 Penaeus vannamei solinvivirus              EM--AFSALLGKIINKTNTKREF----KRATEKQSLPRKDPRNDLSSSC----------- 2266
MZ210029 Fushun monoleptalauta Solinvi-like virus 1 VVLQPYEAYHDDIFHNSAIAALFRQLSTLCQE----------SDFNTSV----------- 2689
KX024775 Nylanderia fulva virus 1                   LALRTFNLTILDLEQNVKSL--ENQEHLEL------------------------------ 2449
KY070327 Diabrotica virgifera virgifera virus 2     LTLRDRLVVQHSIMKAALPKLCDRQETT----------ENLN--NEDKVRRVP------- 2394
OQ540582 Apis mellifera solinvivirus-1              VRIIDRAVIQREIYVEALPYLCS--RHQ----------QNLSINWADEVEREAELTQKEV 2478
MN918666 Picornavirales [unknown host]              VQLTLYETMIEDTFHNVSPLLTFPQKEI----------PKSTTSWADEIETGEPQSAHYL 2190
MW251313 Icha Creek insect virus                    FQYRSQHDIEREVVKMTTKS---LHPEIATPEVPEIPIKDP------------NCLTK-S 2311
MZ822083 Apis picorna-like virus-4                  FCFRTREFLQQQTFEGAYLSGVKRHQGINLVD--------------------------SE 2189
FJ528584 Solenopsis invicta virus-3                 FNFESYKSIQKRYIM---------------------------NGWNEFVKLEKLDLNLNK 2190
MW314636 Myrmica rubra picorna-like virus           FNFRSRLDIQSEIFNGSVMNKVITHG-IDLSEINKIKATSEEDDWNAICAQDTQNETSNT 2093
OL569508 Corparats virus-2                          FSFKSRLDIQEQFYRSALTGLLIRNT-KDIPT--DIPTGKSMHNWTDLVENDPEYSADQS 2538

DQ286292 Rosy apple aphid virus                     DIQKAKISDITNFSVVKDRALAIQK-------------------------YTTTSTTP-- 2131
OP265432 Penaeus vannamei solinvivirus              ------------------------------------------------------------ 2266
MZ210029 Fushun monoleptalauta Solinvi-like virus 1 ---------------------NIIRSGALDNRLNIILKAINSEK-QSVNE-------F-K 2719
KX024775 Nylanderia fulva virus 1                   ----------------PG--------G------Q--------LYFDLLREYYLPSKQF-K 2470
KY070327 Diabrotica virgifera virgifera virus 2     ------SVPRTQD------------------EKPYF------QRY-HQKEYYEQVIKPFK 2423
OQ540582 Apis mellifera solinvivirus-1              E-----AQPITQEQVTENEDLNKTRFGQLTKDTQNF------IDIEHSNAYKRKVLQSLK 2527
MN918666 Picornavirales [unknown host]              L-----N-------------------------YNFF------EKFLFTKTYKRKIMNKAT 2214
MW251313 Icha Creek insect virus                    I-----I----DFGVVAG------S----Q---NFF-------ERKHNRSYYRHIIRFIK 2342
MZ822083 Apis picorna-like virus-4                  L-----Y----EKRIVDK-------------TKEIILR-----YTQTENE-YKLFIEKLN 2221
FJ528584 Solenopsis invicta virus-3                 -------------------------------------KKSSKVTGIHSKQ-YSKFLKFLS 2212
MW314636 Myrmica rubra picorna-like virus           -------------------------------TLDKYY-----QIRNITSD-YSKFLVFLG 2116
OL569508 Corparats virus-2                          I-----S----EHQIVPQLELKTQD----KNLYDKILRFNNRKFRKQGRR-YSELNSLLT 2584

DQ286292 Rosy apple aphid virus                     -ITGSL-------------VHESEMAPLTTNSNPISACLELISKLKTAGPIEHFERTGPP 2177
OP265432 Penaeus vannamei solinvivirus              ----------------------------------PDLTNLILTTSHSTIPCTQVIVDDPP 2292
MZ210029 Fushun monoleptalauta Solinvi-like virus 1 ---MTA-----QPVYRNQNLDAKQLENL-AEKSFISLLNEAWQAGHITKPLYDFW---QS 2767
KX024775 Nylanderia fulva virus 1                   ALRLAL-------------NDQSFFDST-MEFSSVSLLNELFQKGTVSRPKYTVR-LAPS 2515
KY070327 Diabrotica virgifera virgifera virus 2     S-KQSA-------------------MGS-NPKSYISQLNEKFQSGIITILTWVMQ-ENAS 2461
OQ540582 Apis mellifera solinvivirus-1              TIYHNK-------------------LKD-LAMSYRSILNEKFQKGEIGPVHISNK----R 2563
MN918666 Picornavirales [unknown host]              TLSDCE-------------------LNK-LAGAYSQKLNDLYTSGKISKPIIDYTSAGPD 2254
MW251313 Icha Creek insect virus                    AYKGNDADERLEQRDKIWTDKQTNVDYR-MSHSNVGKLNELFQSGRITKPNYSFE----- 2396
MZ822083 Apis picorna-like virus-4                  LLRYKR---------TTDILTYINSEEY-KKMSKFSHLNHLFQRGVLSRPSVDYE----A 2267
FJ528584 Solenopsis invicta virus-3                 RIENEKAAL--EGNFN----KESVNTWY-FKMSKAMHLNEIFQKGLISKPLAEFYF--NE 2263
MW314636 Myrmica rubra picorna-like virus           KLPEKVSLE--EKY-----MNKRSSKYF-IKMSIYSKLNEMFQRGEVTKPELTFM----R 2164
OL569508 Corparats virus-2                          RLPDFVAFS--AEAFASLNLSERNLNNL-KSLSSTMQINELFQNGLISKPCSEYT----S 2637

DQ286292 Rosy apple aphid virus                     H-------NPTYSCTVTYA------------G-------RDFGGEGPSKASSKTQAY-GA 2210
OP265432 Penaeus vannamei solinvivirus              QTSLPNEPTANDLCAEESERVHHPVQQVLLHPIQNCESVHQRVDSGKDNM---PGHAPHA 2349
MZ210029 Fushun monoleptalauta Solinvi-like virus 1 G-------TATWNCLASAA----------KTG--WCQSVKSSNATGVSKQSSKEGAAEKL 2808
KX024775 Nylanderia fulva virus 1                   G-------DISWETTLHLT----------FHEDNSTRSI-TTVGVGRTKSESKEGASFNA 2557
KY070327 Diabrotica virgifera virgifera virus 2     A-------SAKWKAKLSFK----------YTND-ERAPF-EFYGYGNSKTEAKEGAAFEA 2502
OQ540582 Apis mellifera solinvivirus-1              V-------GDEWECEVSTT----------KIA--NSEIF-IAWGRGNSKAEAVEAASHAI 2603
MN918666 Picornavirales [unknown host]              N-------DKTWQCNIICK----------NLA--TCEDL-TAVGQGKTKAEAKEHASYLI 2294
MW251313 Icha Creek insect virus                    Q-------KEFWHCTVTCC----------YDS----RRI-CCEGRGNSKAEAKEGAAFSA 2434
MZ822083 Apis picorna-like virus-4                  I-------GLDWSCKIEVL----------LIS--SKKTL-TGIGKGVSKTIAKEFAAEMI 2307
FJ528584 Solenopsis invicta virus-3                 G-------QKMWDCNITFR----------RSK--DDLPF-TFSGSGTTKACAREQAAEEA 2303
MW314636 Myrmica rubra picorna-like virus           F-------LEEWECEISCK----------DIG--KDRQY-NVSGQGNCKAVAREQAAAGI 2204
OL569508 Corparats virus-2                          I-------QEKWICTITSV----------RQS--DNNSI-KGIGKGVTKAQAREEAAERF 2677
                                                                                                 *  :
```

```
DQ286292 Rosy apple aphid virus                      LRQYLEDHIV--CNAACED----------QRTEALQKIRKV--------AITAFSFYIED 2250
OP265432 Penaeus vannamei solinvivirus               LPKGN------------------------PREFPIPGSRQV-DLSSAYAALTPP------ 2378
MZ210029 Fushun monoleptalauta Solinvi-like virus 1  WYLLFPEDKAGTFRQSLLSQRAVRFASV----------------AGTLTTAQAM------ 2846
KX024775 Nylanderia fulva virus 1                    LKIIGKVPAVF--------------------------------LNGRKKDSAEI------ 2579
KY070327 Diabrotica virgifera virgifera virus 2      YCALFV-------KN------PQRRADLRADK--LQA------------------F---- 2525
OQ540582 Apis mellifera solinvivirus-1               L----------------------------------------------------------- 2604
MN918666 Picornavirales [unknown host]               VSKVDPTTKTLVVKQTLNLDNMRKECDVPPEHVGLQSLSND-ILAGRDVELTIDHFDEKW 2353
MW251313 Icha Creek insect virus                     LQQIPITSSR-TETNTDSDQNDGRTSNQ-----PIPAIRKKIVLQGREKEINPP------ 2482
MZ822083 Apis picorna-like virus-4                   CSQLSKLEN--------------------------------------------------- 2316
FJ528584 Solenopsis invicta virus-3                  LVLFSQEDEI-VR------------------------------------QINDI------ 2320
MW314636 Myrmica rubra picorna-like virus            VELYNSAR------------------------------------------ISDP------ 2216
OL569508 Corparats virus-2                           LEEYVQIK------------------------------------------LQKQ------ 2689

DQ286292 Rosy apple aphid virus                      HINAAIRNAGGKKYIIVIGKR-LADDTVEQVDGFYK----------FHRNGNCYFLSSII 2299
OP265432 Penaeus vannamei solinvivirus               ------------EQETR----------------------------ITSRVSKG-GCLPLE 2397
MZ210029 Fushun monoleptalauta Solinvi-like virus 1  ------------TFDERH----------------------------ESLIEQFWYLVPL- 2865
KX024775 Nylanderia fulva virus 1                    ---------TGVNQSDL-----------------------------N----VCRFCGNY- 2596
KY070327 Diabrotica virgifera virgifera virus 2      ---------RRETYE-------------------------------NTLDKHGDKKVAML 2545
OQ540582 Apis mellifera solinvivirus-1               -------------------------------------------------IKMGYL----- 2610
MN918666 Picornavirales [unknown host]               ERNAISLNINGEEYEAKSRTIKEQKTVYELLDIAYSAIRSDVQEYVDSLSSLGYYIVDI- 2412
MW251313 Icha Creek insect virus                     -----RSTLSGSSYDNF-------------------------------------YN-PSS 2499
MZ822083 Apis picorna-like virus-4                   -------------ERSR-------------------------------------FI---- 2322
FJ528584 Solenopsis invicta virus-3                  ------------QSDCK-------------------------------------FC---- 2327
MW314636 Myrmica rubra picorna-like virus            ------------NTSVR-------------------------------------F----- 2222
OL569508 Corparats virus-2                           ------------SY---------------------------------------------- 2691

DQ286292 Rosy apple aphid virus                      EKL----------------------------------------GRKTAAQ---------- 2309
OP265432 Penaeus vannamei solinvivirus               GSSTRGS------------------------------------DS--ARSNMPASSPRSA 2419
MZ210029 Fushun monoleptalauta Solinvi-like virus 1  -----IYL-----------------LYLVIRKTIKYVSIGNGEIK--YDQELP-VHYMSQ 2900
KX024775 Nylanderia fulva virus 1                    ----YDESRYPQGCPCNKENRPNNADYWEDL----TIPIRDGERPMTFRE---------- 2638
KY070327 Diabrotica virgifera virgifera virus 2      DKCVATSPIAPTTCSCK------------------CNRID---ER--------------- 2569
OQ540582 Apis mellifera solinvivirus-1               --------------------------------------------K--------------- 2611
MN918666 Picornavirales [unknown host]               GK-PQHYPLLPQVKSLVDLLVDQKISYFDITHYSEYIEIEAGNEKKTYRQAIA-ILPREE 2470
MW251313 Icha Creek insect virus                     SRSDVHYPTVITP------------------------SI----ERS----------VRSN 2521
MZ822083 Apis picorna-like virus-4                   -KQ--------------------------------------------------------- 2324
FJ528584 Solenopsis invicta virus-3                  -KKMIRY----------------------------------------------------- 2333
MW314636 Myrmica rubra picorna-like virus            ------M----------------------------------------------------- 2223
OL569508 Corparats virus-2                           ------------------------------------------------------------ 2691

DQ286292 Rosy apple aphid virus                      ----------CYSYYPGVSFDSSTNEVTL-------DVHASTSNYSLR--EDFEKDYTDD 2350
OP265432 Penaeus vannamei solinvivirus               PLN-SKQRRLKRRAEERAALAAENATK----------QSSAELPDAPRRFTPSPAE-RQR 2467
MZ210029 Fushun monoleptalauta Solinvi-like virus 1  ---KEFNRRLRRSHYLRKQLAQAIAKVQQHNRQSDLRKQAGDMNVS-------------- 2943
KX024775 Nylanderia fulva virus 1                    -----------IKDFPRLAME--IDYV-------PTKLTREEMEYIEDLLDSVAGENQND 2678
KY070327 Diabrotica virgifera virgifera virus 2      ----TPTRNSKFRVKSKMALN----------------NFRQEMNVT-------------- 2595
OQ540582 Apis mellifera solinvivirus-1               ----QQKKMAPQ---MRANLN----------------HFVQEMNVA-------------- 2634
MN918666 Picornavirales [unknown host]               VFSTLLDRIIAQIRAPPLDLD----------------QMTQEMNVS-------------- 2500
MW251313 Icha Creek insect virus                     ----D----FQRDLFNAVEQE----------------RALRERNQSKRRRIDLDGRYQKE 2557
MZ822083 Apis picorna-like virus-4                   ---------------SMPFME----------------P---QMNVS-------------- 2336
FJ528584 Solenopsis invicta virus-3                  -----------KKLLSGVSIQ----------------R---QMNVS-------------- 2349
MW314636 Myrmica rubra picorna-like virus            -----------RQMLLKTKFE----------------R---QMNVS-------------- 2239
OL569508 Corparats virus-2                           -----------RTLFPRLGLK----------------L---ESNIS-------------- 2707

DQ286292 Rosy apple aphid virus                      DHLIIANSAEDKPT-------------------L-GVMANDDPVTMPVTSVMDS----DK 2386
OP265432 Penaeus vannamei solinvivirus               ---LMKKATGALPTAWDKLRVTPLPESPAEKQSLGAPSADAVPQMESGAPAGGNGPAPAV 2524
MZ210029 Fushun monoleptalauta Solinvi-like virus 1  ------GLQLAT-----------------------PAPSGVDVNTTTGTSIFTNRMIGNT 2974
KX024775 Nylanderia fulva virus 1                    ---IPLSTEGNVPT---------------------E-ARQTEPTVTSGNMLWTNVGGKFG 2713
KY070327 Diabrotica virgifera virgifera virus 2      -----PQSVGAIPQ---------------------APMTQTDVSVVTDSGLFTNTNAPSP 2629
OQ540582 Apis mellifera solinvivirus-1               -----PQVSNVVPK---------------------APFLASDVSVVPDAGLYTNTDAPAI 2668
MN918666 Picornavirales [unknown host]               -----AGTVGKIPT---------------------APMTQTDVSVVADSGLYTNTVAPSP 2534
MW251313 Icha Creek insect virus                     ---MMLTPHMGVTT---------------------GQISSLDPTVVPQQIQATNTTFPTS 2593
MZ822083 Apis picorna-like virus-4                   -----ASLEHKTLG---------------------APMIAPDSTVTPDSGLVTNTMTPSP 2370
FJ528584 Solenopsis invicta virus-3                  -----KITENHVPS---------------------AGMMATDPSVAPDSGIATNTQTPSI 2383
MW314636 Myrmica rubra picorna-like virus            -----KGVEHAVPG---------------------ARMVSTDPSISPDTGIATNVDTVSP 2273
OL569508 Corparats virus-2                           -----AGVEHATPG---------------------APMISADPSIAPDTGIATNTMTVSP 2741

                                                                                             Jelly-roll fold
                                                                                             capsid protein domain
                                                                                             AmSV1: 2705-2864
                                                                                             ____________________
DQ286292 Rosy apple aphid virus                      ALYQPIIMNRSAPNNSWLRAGGITFNIYDLVYNQFVGC-NKQITVGDGTAAGTILAQIPY 2445
OP265432 Penaeus vannamei solinvivirus               GPVHPPSLLQGMIT---LAG--TAQNLLNSAMEFV---MGTPLQIDPSIAAWTVIKEISL 2576
MZ210029 Fushun monoleptalauta Solinvi-like virus 1  RLT----LNNTTSVINNPGAAGAPYDYLTKIHGIYS--LHLSGTLSSTTASGAEVFRVSL 3028
KX024775 Nylanderia fulva virus 1                    YMM----TNSESSALTNPRGTGAPFNYLRSLFEIYR--PEIRTTINGSIARGSLLAVLSL 2767
KY070327 Diabrotica virgifera virgifera virus 2      AKI----INSVGIALDNPAGSGAPFNKHVSVYNIYQRWEAKNTVLSPALPSGTEIVRISL 2685
OQ540582 Apis mellifera solinvivirus-1               AQD----VNHTAIALDNPAGTGAAFSKHQSCYQIYQNWDEKNTTVNPSMANGTEIMRLTL 2724
MN918666 Picornavirales [unknown host]               AKV----MNSLGIALDNPAGTGAPFNKHVAVYNIYQRWESKNTSISPSMSQGTEVLRISL 2590
MW251313 Icha Creek insect virus                     VPD----FNPGAVAINNPAGTGAPFDKKESVYNIFQEWESKNTSVNGTVPRGAIVCRISL 2649
MZ822083 Apis picorna-like virus-4                   CRV----INPGAISLDNPAGTGAAFDKRDSIYGIYQRWPEKSAIVNGSLQEGAEVVRISL 2426
FJ528584 Solenopsis invicta virus-3                  SRV----LNPIARALDNPAGTGAPFDKHTYVYNVFTRWPEMSTVVNKSLAAGAEVFKISL 2439
MW314636 Myrmica rubra picorna-like virus            VRV----INPGAIALDNPAGSGAAFDKRDSIYRVYHRWSEKNSTINGSLTQGAEVLRISL 2329
OL569508 Corparats virus-2                           ARV----INPLAQALDNPAGTGAAFDKKDAIYGIYTRWTEKQGTINGSLNQGAEVFRLSL 2797
                                                                        .     .                  :.      : :  :
```

```
DQ286292 Rosy apple aphid virus                        DPVDNPFANGYIQNLVLLHGRMTGDWMIKITCPGNPGMQCSIGVGWSPSK-IAETTVPMD 2504
OP265432 Penaeus vannamei solinvivirus                 HESKWQN--PVARLFYQLHKNRSPAIDIKVEVTGAVFDVGKLATCIVYGPPANGEKYSDL 2634
MZ210029 Fushun monoleptalauta Solinvi-like virus 1    DPATWPE--E-LQTYVNMHESICPDIDISVMINGAAGALGTMKIGWVPDA-DAKNKYSLA 3084
KX024775 Nylanderia fulva virus 1                      NPANYEP--Y-QALWAKLHRYFSPQRCFLVNLAGAAGSIGWVTFSWHPDE----EDLRFE 2820
KY070327 Diabrotica virgifera virgifera virus 2        DPKELPQ--M-IRSYIDFHESFIPALDIVIAIAGAAGTIGWIATGWVKDA-SSTKKYTLQ 2741
OQ540582 Apis mellifera solinvivirus-1                 DPKTWPK--R-IVDYIMFHDSILPALDIIVTIAGAAGTIGWIKSGWVKDA-SSTKKYTSG 2780
MN918666 Picornavirales [unknown host]                 DPRDLPD--M-IQSYMQFHDSFIPAIDVTIAIAGAAGTIGWIATGWVKDA-SPEKKYSLS 2646
MW251313 Icha Creek insect virus                       DPQYYPR--K-ILEYILFHQQIIPEIEVGLAIGGAAGTISWLRIGWVQDA-A-KEKITLD 2704
MZ822083 Apis picorna-like virus-4                     DPKTLPA--R-IREYVNFHEYIIPAIDVQIVIGGAAGTIGWLKIGWVPDD---TLKYGLD 2480
FJ528584 Solenopsis invicta virus-3                    DPNKLPK--R-ILQYIQFHKTIIPQIEVQILIGGAAGTVGWLKVGWVPDA-STAKKYSLD 2495
MW314636 Myrmica rubra picorna-like virus              DPRSLPK--R-IREYIDFHNAIIPQIDVQILIGGAAGSIGWLKFGWIPND---AMKVTLD 2383
OL569508 Corparats virus-2                             NPQTLPQ--R-IREYIAFHQSLIPQIDVQILIGGAAGSIGWLKLGWVPDD---SKTYTLD 2851
                                                       .                 :*      . :    *       :        .

DQ286292 Rosy apple aphid virus                        VITKYAFFTTGVSSEWSHTIVMTDARQNNFYRTIQRNSKKTEVEDVANMPHIVIFCETPP 2564
OP265432 Penaeus vannamei solinvivirus                 ELQQHYLTLYATNENNTVVTTLTDRRTSHFARTD-E----YDREPESQYPKLLVFVAVPI 2689
MZ210029 Fushun monoleptalauta Solinvi-like virus 1    DLQSIASTDVTFNQTSVFEIGISDLRRANQYRKT--------VGDGEPFPGVVALISLPV 3136
KX024775 Nylanderia fulva virus 1                      DIDEVTSWTTNLNNHQCMEFILDDARIIQQMRPTAN----PTMNGTEKFPCIAMWVQEPA 2876
KY070327 Diabrotica virgifera virgifera virus 2        DLQQVAMEESNMNGTQIIKIKLTDVRRMGLYRRV--------TDDPEPFPGIIMMVDHAV 2793
OQ540582 Apis mellifera solinvivirus-1                 DLQQIESKVVNMNGTQVMKFTLNDVRRSGLYRLI--------ENDPEPYPGIVFIVESAV 2832
MN918666 Picornavirales [unknown host]                 DLQQVSLEEANMNGTQVIRVLLNDIRRQGLYRKM--------LQDPEPYPGIIMMIDHAV 2698
MW251313 Icha Creek insect virus                       EIQQISMQQINMNGSTMTTFPLRDAMRRSFWRSV--------KNDPDPYPGIVMVAHPA 2756
MZ822083 Apis picorna-like virus-4                     DLQAVACETVNMNGTITMCTLLTDNRQYGMYRKV--------QNDSEPWPALIMVDHPA 2532
FJ528584 Solenopsis invicta virus-3                    DLQLVASETINLNSTITMSMIINDSRRNGMFRLT--------KSDPEPWPGIVCLVEHPI 2547
MW314636 Myrmica rubra picorna-like virus              DLQLVAAEEVNLNNTLTMCTMLNDNRRSGLYRLV--------KDDPEPWPALVLMVDHPA 2435
OL569508 Corparats virus-2                             DLQLVAAEELNLNNTLTMQCILNDNRKNGMYRYV--------DNDPEPWPALILMIDHPA 2903
                                                        :          .         : *        *                    * :

DQ286292 Rosy apple aphid virus                        TSVYTEKKSTYLTFFSKLCSESDLMFNPSIKPFVLA----DPQQSVTYD---------SK 2611
OP265432 Penaeus vannamei solinvivirus                 YNTSGADTPRIRIRVGDRLHPSSIFTMPNLPLVRSLT----------------SSGTGMH 2733
MZ210029 Fushun monoleptalauta Solinvi-like virus 1    GNVQKTGTIEFGWQVYMKLGANCFFMNPKSVTTTK----------------------VN 3173
KX024775 Nylanderia fulva virus 1                      TNVQRNNDVSYPIQIRTKLGPNAVFVTPSRAGIEGG----------------SGGGGVF 2919
KY070327 Diabrotica virgifera virgifera virus 2        TNVQRNDAVNYPVRVQVRLDQTCILMEPFNLPSSSP---------------------AT 2831
OQ540582 Apis mellifera solinvivirus-1                 TNVQRNDAVNYPIRVQAKLNPACLLMEPFRSSGSGP---------------------SV 2870
MN918666 Picornavirales [unknown host]                 TNVQRNDGVSYPVRVQVRLAPNCVLMEPFNLPSSIN---------------------PT 2736
MW251313 Icha Creek insect virus                       TNVQRNDDVDYPIQVFMRLGPGCQLMAPLNDLTTGD---------------------DS 2794
MZ822083 Apis picorna-like virus-4                     LNVQRNDDVGYPVRVNVRLAPTCALMYPNRLISGAP--------------------EVG 2571
FJ528584 Solenopsis invicta virus-3                    TNVQRNDDVNYPVIVSVRLGPDCQLMQPYNDFKLSGGTDPDPDPEPDPDPEPGPDPEPGV 2607
MW314636 Myrmica rubra picorna-like virus              LNVQRNDDVNYPIFVNVRLGPNCLLMQPYNTITNII--------------------DGT 2474
OL569508 Corparats virus-2                             LNVQRNDDVNYPVFVNVRLGPQCCLMQPYNMITTEL--------------------SSG 2942
                                                        ..            .          :  *

DQ286292 Rosy apple aphid virus                        KGY---------SLNFLLAPQL----STR---L-----RGAS---------LGNIFGLAY 2641
OP265432 Penaeus vannamei solinvivirus                 TTIPHA---YH-HLKIVIDGAYYISSNER--RHLGPDINGYLV--------TGPCVPVDE 2779
MZ210029 Fushun monoleptalauta Solinvi-like virus 1    EKINLDEHITDEIIDFLVGSDGIAATATD---------QTYDTGVTAL----KNAIPIGS 3220
KX024775 Nylanderia fulva virus 1                      NSISLSNYIPT-NFDTVVGDAGVPQAYS---VVSLPD-RGYLTGNFDFNSDEGNVVGYRR 2974
KY070327 Diabrotica virgifera virgifera virus 2        TSFDLGSYFYDTHVDALIGSSAVADDPES--VVVHPD-SGFNVKDFSPVFGHNCVAAIY- 2887
OQ540582 Apis mellifera solinvivirus-1                 IDFPIGSYFYDDAVDMLVLGSSVPNKQPT--ENDIDD-QGWLKGDFQPTFGAKNVIGMSW 2927
MN918666 Picornavirales [unknown host]                 QSFNLGSYFYDDDVDALVASSALTSSPTA--TTTFPD-CGYLHKDFQPEYLQSNIYTVAY 2793
MW251313 Icha Creek insect virus                       TAIDLEPLLGGVKPDLVETVQAQPQ-LV--KTVEFPD-CGFHTGTFQPIFRTANIHAMNA 2850
MZ822083 Apis picorna-like virus-4                     TKLSLKPFLNASTVDFLIGSSKILNEGIS--LLEYPE-CGYLSGNFNPTFTDRNCINAHL 2628
FJ528584 Solenopsis invicta virus-3                    DELDLSKYIPNQLIDLLICNSYVPNNVSVDFLSTYPN-LNFS---------IHNITDVVV 2657
MW314636 Myrmica rubra picorna-like virus              TRIALSGILQGQSIDLLIGTPNPCNRPTS--TKAYPD-CGWYTGQFQPEFTRDNLCSVGM 2531
OL569508 Corparats virus-2                             TIIDLNRYLKLNSFDLLIGTSSICDNVVQ--PHSFPD-CGFLTGDFKPKFSKENWCGTFQ 2999
                                                                     . :

DQ286292 Rosy apple aphid virus                        TDYIY-------VHIEGDK-----------------------LT-----------D--- 2656
OP265432 Penaeus vannamei solinvivirus                 RPHAG------------------------------AIRYSYVN---------PGTH--- 2796
MZ210029 Fushun monoleptalauta Solinvi-like virus 1    IMENG---------YYVDGSLSADE-ISNLKQED--FTWYYQDPSDSNKVLTKSYHTKPK 3268
KX024775 Nylanderia fulva virus 1                      FNAEGAYRFVKAVHYYGESPDTFWTKPVVDVKIVPETARFDREE---------HGLSVSQ 3025
KY070327 Diabrotica virgifera virgifera virus 2        KQLDGATKAVPTI-SNDTTPSA--D---YLKEIED----MDKFE----------GGPNAQ 2927
OQ540582 Apis mellifera solinvivirus-1                 NEDMLDAPNKKLV-IVTELPELTPE---IITDFGA----FKEMD---------DDLTTQI 2970
MN918666 Picornavirales [unknown host]                 NALAKGKTAIQLF-TTEDTPGAT-----LLAQLDS----LTFTP---------SASDQLS 2834
MW251313 Icha Creek insect virus                       LECHF-------T-------SNVLE---NVTQENV-----DSAP---------EGSLKGF 2879
MZ822083 Apis picorna-like virus-4                     RRTNY-------I-VFTDN-LSKI-------------TLGTKIA---------QYSTQKF 2657
FJ528584 Solenopsis invicta virus-3                    SSKPY-------------T-LALFE---TESQINSASVWRGDLT---------QLSVFIQ 2691
MW314636 Myrmica rubra picorna-like virus              SWTSG-------V-IVT-R-EAAYE---V--K--YPLTIKG-----------TGTGERY 2562
OL569508 Corparats virus-2                             QAGTV-------I-YTTLS-QSAFQ---T--QVDSGIIIKD-FD---------YASVSSI 3035

DQ286292 Rosy apple aphid virus                        ---LIFQNALTNIPDFTTIS-LF-QHEISSTETNYTFQFCSVGYA-----SYY------- 2699
OP265432 Penaeus vannamei solinvivirus                 ---THFYPFYGAMTLDE-ASAIYQDELSVVYDASDDIMSFEAEIG--------------- 2837
MZ210029 Fushun monoleptalauta Solinvi-like virus 1    AIRSYLLYAFGVIPDIMLPA----NQVIDLQGILCNIASIDLGFS-------QPITQCVL 3317
KX024775 Nylanderia fulva virus 1                      SPISHIYIAYGEVPTYDYGGV--EDPFSLMPGTIYTIPPTDVTFSKGGRSIFGRIMRVMT 3083
KY070327 Diabrotica virgifera virgifera virus 2        LMPNKLIFSFGETAPLE--DFHYVNAEPTLLGSTYALSG-AVRVG-IFK---YNITSLVA 2980
OQ540582 Apis mellifera solinvivirus-1                 IPPNRIFYGFGKLPVEK--FMDYNGKKDMPGATVYKTNF-SVEIG-NLK---YGNSTTFI 3023
MN918666 Picornavirales [unknown host]                 LLPSKVYFAFGEVETSD--HVYYEEEEPTIQGTVYTISD-TTRLG-LGF---YNCRTLIA 2887
MW251313 Icha Creek insect virus                       QHVWQIIFSYGTVPDQDFNHLSHDNASLNIPASQATEFVSDITMG-SII---YQNTQLWV 2935
MZ822083 Apis picorna-like virus-4                     EFPSSIDFAYGQIPSVFERMTIYPDAKF-GSFSNYTIQPLFITNG-VFI---YKCTTMYV 2712
FJ528584 Solenopsis invicta virus-3                    YKFYTRVEAYNKVTTVHTDKW-----TPNFDGTVYKPVDVKIEHAYG-T---YELTTMWL 2742
MW314636 Myrmica rubra picorna-like virus              YRPYSFVFAYGEVDASICERYTYRGEDPPPTGTTYTIPKFTIKFG-SMR---LSCTQVNA 2618
OL569508 Corparats virus-2                             TIPGGIVFGYGNIPEAYTGMSTRVSDALQFNSTKYTIPKYTFRTSSGLE---YACSQIEM 3092
                                                                                                  .
```

```
DQ286292 Rosy apple aphid virus                        QFNGYFVVFQSNSGNFLPLIKD-----YTSSS------E-YADIRRTI------------ 2735
OP265432 Penaeus vannamei solinvivirus                 ----DILDV--------------------------------------------------- 2842
MZ210029 Fushun monoleptalauta Solinvi-like virus 1    YEHGYILLQPVGTPTITTSGLNVRVVDYEKDPIYYGWVANLTNVSQTTVCGYKENACCLP 3377
KX024775 Nylanderia fulva virus 1                      FEKGLIAETKIGNLAGINDI---------------TWVPVASQ--------------KTG 3114
KY070327 Diabrotica virgifera virgifera virus 2        QANGCYIVMEIDSSVVDSTKHGK-------------LVVYNSCNNTTNFDQ--TD----- 3020
OQ540582 Apis mellifera solinvivirus-1                 YPEGCVIMLEARYALHRTEIGNIPDC---------IAVVYLDSVKKNTFEFYQDTQQTLN 3074
MN918666 Picornavirales [unknown host]                 QASGCIIVAELDHSVIEQSG-----------------KRYISYNKMNT--------DVV- 2921
MW251313 Icha Creek insect virus                       YPNGISLKFNSLPKDFANGIRGIPSV---------RFSD-LNIG-------------AWP 2972
MZ822083 Apis picorna-like virus-4                     FESGCILHFSIDGNYWEIT-VS--------------------GSEITTIGQ------AYE 2745
FJ528584 Solenopsis invicta virus-3                    TSYGLVMEWSLDESRVFYGTYKTDS-----------------NGRRWLIDG------NTP 2779
MW314636 Myrmica rubra picorna-like virus              FENGIVMVFRCDNVQRIHCQIES-------------------NGDITMLGD------AQD 2653
OL569508 Corparats virus-2                             FESGCIMYFLLDNVVVYNISYSNNNT---------QFTL-TSKGDRNSFGQ------TIN 3136

DQ286292 Rosy apple aphid virus                        ----YDWYNPNYVEDDYCTFLKVNQVNKMFIEKPVSCVAH-TTFSG----------SK-- 2778
OP265432 Penaeus vannamei solinvivirus                 -----------------------------------------HQFNGVIKYGTYADTRPCY 2861
MZ210029 Fushun monoleptalauta Solinvi-like virus 1    -----YG-----FELS------ELPVQLASNEFLNNFDA--------------------- 3400
KX024775 Nylanderia fulva virus 1                      SVIYYLWDG---VVNG------ANPV-----SDLFGELSGGP---------------SSL 3145
KY070327 Diabrotica virgifera virgifera virus 2        I-----------DH-------------------AIVYDTR-------------------- 3030
OQ540582 Apis mellifera solinvivirus-1                 ISTFTYWNREQTHH-------------------LFDPVIG-------------------- 3095
MN918666 Picornavirales [unknown host]                 --------TQSDLD-------------------SFGPDVG-------------------- 2934
MW251313 Icha Creek insect virus                       --------------------------------------------------------RAEE 2976
MZ822083 Apis picorna-like virus-4                     PD-SYYWGTYNMLVAK------TTDANVVYNKTPLDFDSLNV-FKGQFIYSTY---FGGK 2794
FJ528584 Solenopsis invicta virus-3                    IARSDHCFIVS--SPD------LLSDDKAYYNNPIGAKQGGKLVDGAQIYRIFKTESGGY 2831
MW314636 Myrmica rubra picorna-like virus              IT--DYVFLYT-VDKA------E------QFVNPLDFTSA-------------------- 2678
OL569508 Corparats virus-2                             VA-----FLQNYMDPG------KLPISMIDLSTPIGF----------------------- 3162

DQ286292 Rosy apple aphid virus                        --------SEWVFVPDSLEVIE--FIKDRVKFR--------ITKLL--LPDNNDRKMSFK 2818
OP265432 Penaeus vannamei solinvivirus                 WVRTTRATFLAVQ-----PKNGQQYNT--------------------T----------VR 2886
MZ210029 Fushun monoleptalauta Solinvi-like virus 1    -RKQNVQ-HNWLQ--------GTGYVENGFKYCSQKSTDNSFQPWVP-NSSDTLEDIKIP 3449
KX024775 Nylanderia fulva virus 1                      AGNGNVVPTGWPTGGVTGFQVGD------------------FARW--------------- 3172
KY070327 Diabrotica virgifera virgifera virus 2        -KEVEVN-WIFQEFSHNKIASG--------------------NPFRMPVRIDQTSEIYVT 3068
OQ540582 Apis mellifera solinvivirus-1                 -KIGNLG-PDWKEL--------------------------------------------VD 3109
MN918666 Picornavirales [unknown host]                 -QDTKVS-ILWRDPNHNRDVVG--------------------DDLLVRHPIATT-THYVV 2971
MW251313 Icha Creek insect virus                       WVPQNVDIYCWTGSEWGRHVGGTSYVP-NN---------NTGNQFTLTCPTT-------- 3018
MZ822083 Apis picorna-like virus-4                     WQRALASY-------INLPIDEN---IDGF---------TDRKKWF------------LP 2823
FJ528584 Solenopsis invicta virus-3                    RSDPFVPETYWP---SETPYNAD------------------------------------- 2851
MW314636 Myrmica rubra picorna-like virus              -----TPSYWWSITPTNTPISHNNQNLMPR---------TRPKDFYFAVPY--------- 2715
OL569508 Corparats virus-2                             -----QPLTCWEYRSSSQIFSITSSV---L---------TFSRRYYIAVARDDTR-ISFD 3204

DQ286292 Rosy apple aphid virus                        NLKSFDLNYPPINQIEKALNDVPLANLPQGIRQVVFSSYPQ---LSVNNPFRGRQ-TPHI 2874
OP265432 Penaeus vannamei solinvivirus                 NI---TGM--SGLLPIQM--SAKDPLTPTGESRCLFQQHSSSITSGNYAGENSLPFAGTL 2939
MZ210029 Fushun monoleptalauta Solinvi-like virus 1    FK--VEDG--YVFAPSAVSSNGSTITTSTGLTEFFFAKPGFSLTNKINS-YIPPL-ASGV 3503
KX024775 Nylanderia fulva virus 1                      SVTRQSRI--LNLTFLQTGNNAATEVLPEGLRAFYFMRPGTSTNLTSGSGFFGPL-VPNA 3229
KY070327 Diabrotica virgifera virgifera virus 2        DT--QSDK--KPTVYVQTGNSMPSTTLPTGLKQLSFVRSGTTTQVTEDLPFVPLY-KPII 3123
OQ540582 Apis mellifera solinvivirus-1                 DIKPTVKE--VTKTFLCTSNTAPSTILPTGLKQISFVRKGTSTSDPHDNPSNVPE-KPIL 3166
MN918666 Picornavirales [unknown host]                 NPSTIRSS--VLQTFVQTGNSMPATNLPAGLKQLTFVRSGTTSTVSTDLPFVPLE-KPML 3028
MW251313 Icha Creek insect virus                       ---DMPVT--DIPNYVFTGNSFPFISLPDGLKAFYIARSGQTTS--VDSPINVVA-ARGI 3070
MZ822083 Apis picorna-like virus-4                     DQKRTTTL--YDWTYVLTGNSLPATALPNGLKTAFMVRPGTSTTTADDAAFAPIT-APGI 2880
FJ528584 Solenopsis invicta virus-3                    WSGVKMPY--QIRKVIQTGNNLAGKHLDGDLKMCAMIRQGSSSTQSTDNYFYPIY-VHNF 2908
MW314636 Myrmica rubra picorna-like virus              PGVKDNRF--FQFTYIQSGNNVPTTSLPAGIRFAAMCRPGTSSNISNESGFYPLL-APGI 2772
OL569508 Corparats virus-2                             NAEQADRL--YVWSFIQTGNTAPATALPNQIKVCSLIRPGTTTTQVTDNGFYPMI-VPGL 3261
                                                                                                  :

DQ286292 Rosy apple aphid virus                        APIFLFISRLKQQYGSETVE---IVFN------SAFNGQ-QVI---VARIDLSTAVE--- 2918
OP265432 Penaeus vannamei solinvivirus                 DYTFAQEFGIEDDEAVVAL------TDRNTDSTVCYLRWSGTFKSWSIRATPYLAS--NF 2991
MZ210029 Fushun monoleptalauta Solinvi-like virus 1    VTLLKRLMNLANG---RIM-NFTIFIQDMPDFQVTLNG--ELI---LIKPTEVCMAKRGV 3554
KX024775 Nylanderia fulva virus 1                      WGLLDRLQDIADQLQTNILHFSWVGADGVEIGKLGYSS--GFI---VCRCSTSRLIR-AG 3283
KY070327 Diabrotica virgifera virgifera virus 2        KNAFRAFDEYLVNNGFSNLVRGDLYIAGENKGQIGYQN--STF---FVRTDDFKKIF-AN 3177
OQ540582 Apis mellifera solinvivirus-1                 KGVILGLEQVLQQLGTNGL-VGNLEINGENIGQVGFAD--ETL---IVRTDDFKVIK-VN 3219
MN918666 Picornavirales [unknown host]                 PNAIAALNKVLSDNGWKAL-KADLYVQNLNLGQVGYQD--GYF---FIKSDLYRQIL-PR 3081
MW251313 Icha Creek insect virus                       NNVFDKLDRVAQDYQVTWL-KMNLIVDDNIIGQLAYAN--RTF---ICRTNDVLRVR-AS 3123
MZ822083 Apis picorna-like virus-4                     HSLYKTLNDYLVTLNTELI-IADLVVAGNILGKVGYVK--NAF---LTRTSGVKVIK-TA 2933
FJ528584 Solenopsis invicta virus-3                    SALLKQMNLILKERKTKYI-KFDLQVGGKPFAQMGFGD--GAF---IGRTTMFRQIR-AA 2961
MW314636 Myrmica rubra picorna-like virus              AGVFGNLDLIAHSLKADII-QMDVLVDGRLFTKVAYAD--GTF---LVRTNVSRRIR-TA 2825
OL569508 Corparats virus-2                             EVVYTQLDQIMDNLGANLL-QFSVLVQGQVIARLAYTQ--GAF---LGRTSSIRLIR-TA 3314
                                                                         :                       :     :

DQ286292 Rosy apple aphid virus                        -NTFMYIK---TED---DAPLYSTLRFPSAMLELLSIQTVYNGTTQ-----AF-SDASEF 2965
OP265432 Penaeus vannamei solinvivirus                 SLSNCYVSHITRE-FSGKLPDTDLSNFSSRV--------ASQGTPQ---------KIV-- 3031
MZ210029 Fushun monoleptalauta Solinvi-like virus 1    RVSETYLSNFTFTSNIETLQTFNTTQ-IPWT--------TIVATSQ--------SKTF-- 3595
KX024775 Nylanderia fulva virus 1                      WVPNSKFVDIEAATSFSALPSLPIASYVSWK--------TNTQL----------GVTRNF 3325
KY070327 Diabrotica virgifera virgifera virus 2        IGQDIKIKNIADLPQANAINAFNTTGMSTWI--------SSGKRTR-------R----RL 3218
OQ540582 Apis mellifera solinvivirus-1                 VGTNVVLRNIVGITDLKGITQFNTKGFSSWV--------SQGLKKT-------KARRFNL 3264
MN918666 Picornavirales [unknown host]                 ITTACILQNVTQLDNVQGLTTFNLSSFVSWV--------SASKRL--------RHR--QL 3123
MW251313 Icha Creek insect virus                       LTTAMSLTNIEPVDNPNTITPMNTNTWSPFK--------QVPTTLSLVLPKAAIKRLPEE 3175
MZ822083 Apis picorna-like virus-4                     LTTDIQMINITAASNTSAISVLNVGGFASWS--------GSNT--------YFVGRVFSF 2977
FJ528584 Solenopsis invicta virus-3                    ITNVILLKNIVGVDDLSGLQALPTSGFADWV--------VKAQ--------STNSKFLND 3005
MW314636 Myrmica rubra picorna-like virus              LSTNIILDNIQPIDNINTLPPVPINGFEPWT--------TGAA--------SLSDRFMNM 2869
OL569508 Corparats virus-2                             LTKDIRLTEIARLDNINVLPLLPTQGFSSWN--------SSSSQ-------SFRSKVVDF 3359
                                                             .
```

```
DQ286292 Rosy apple aphid virus                       DS------NVITANAGAAAAFIGGGLLSGLG-------QGLGAYAQGKMQMQMQDKTLSW 3012
OP265432 Penaeus vannamei solinvivirus                -------RCRTDLTE--PPAERQGNLISTSMQIHAQQNMLTQQLEQQAWQNELNREYNAW 3082
MZ210029 Fushun monoleptalauta Solinvi-like virus 1  KS------RKMKQSGAFWG-MAAGSMMSGLG-------GGMSAYFANQQDQQNLREIMDT 3641
KX024775 Nylanderia fulva virus 1                     FVRPQKPISGINQSA---ALAIGGGLLSGLG-------SGLGAVYASKRQSKLNAENW-- 3373
KY070327 Diabrotica virgifera virgifera virus 2       DL------N-NFHQQ---SATVGGAVMTGLG-------NGFSAWGDQLFQ---------- 3251
OQ540582 Apis mellifera solinvivirus-1                NL------C-QYES-------FAGIAASGIG-------ALMGSVGQNVMNQYNMDKIMEQ 3303
MN918666 Picornavirales [unknown host]                NL------D-NYTRE---ASAIAGGAISGLG-------QGITAWSNQMFQ---------- 3156
MW251313 Icha Creek insect virus                      TFRRKIAIAGKYQRESAIVA-GLGGAMTGLD-------NYLSQQ-----------RNFTQ 3216
MZ822083 Apis picorna-like virus-4                    Q---------SAFMALGMAAQAGSNMMQAIG-------NGLMYWDYKSWQDARLDKQNAL 3021
FJ528584 Solenopsis invicta virus-3                   FYNDKISIERQASLGIAAAIGAGQGLFGGLS-------AQWQWQQQADWSRQMQRERLDM 3058
MW314636 Myrmica rubra picorna-like virus             KF--------MRQSAALIGASAAGGLFSGLT-------SALQFGEYNQWRNNYLQKQIDA 2914
OL569508 Corparats virus-2                            LF--------QKQSGAFMAVGALGGLFNGLA-------NTMQFQEYNEWKDAYQKRHLDT 3404

DQ286292 Rosy apple aphid virus                       YEQKQ-----------KHDIDNQRYLQQSMFDWKSHMQQRDFDYGNEQQRRQIDWQRSSQ 3061
OP265432 Penaeus vannamei solinvivirus                ----------------NAQQD-------RQHSEFLQTRDQHFKA---------------- 3103
MZ210029 Fushun monoleptalauta Solinvi-like virus 1  NNKFK-------KQYLAQQFDQQLAL---------QTQQQNFQR---------------- 3669
KX024775 Nylanderia fulva virus 1                     ----------------HHLSDLQHSLLGIKNSQELAKMDKQFQQ---------------- 3401
KY070327 Diabrotica virgifera virgifera virus 2       -ERMQ-----------NAVLANNRAIAGANNAALAERQKAAFER---------------- 3283
OQ540582 Apis mellifera solinvivirus-1                QKLMQ-----------SMQLSNQRTIAEANNSAILARQQKAFEN---------------- 3336
MN918666 Picornavirales [unknown host]                EN-MM-----------KQLMANQQALQGANNAAIAKRQQEAFDR---------------- 3188
MW251313 Icha Creek insect virus                      QKDLV-----------NLQGDNQIRVLQARYAQQKDYQAYLNHL---------------- 3249
MZ822083 Apis picorna-like virus-4                    RLQLMEMQNEAAKQVQAMRNQNEIDK-----------IQKQAEI---------------- 3054
FJ528584 Solenopsis invicta virus-3                   MEKLA-----------NINNQARLNQLTQSGAQQRITQQAAYQQ---------------- 3091
MW314636 Myrmica rubra picorna-like virus             QRSLA-----------MMNNQNQRDL-----------SKQNFDQ---------------- 2936
OL569508 Corparats virus-2                            LKQLE-----------GIRGENQLNL-----------SKQNFDQ---------------- 3426

DQ286292 Rosy apple aphid virus                       AALFSQQNLYQDTQNKFTKELVETNLDSDIRRARNAQQLSGYRTDATVSGLSNSGRG--G 3119
OP265432 Penaeus vannamei solinvivirus                -----------------------------------QMRGLR-------LGNLDGRDIPAG 3121
MZ210029 Fushun monoleptalauta Solinvi-like virus 1  -----------------------------------G--NIGLSNGGAVGNMMRGNTASAS 3692
KX024775 Nylanderia fulva virus 1                     -----------------------------------QLELIGKSSYSAMSGNTYGPRG--E 3424
KY070327 Diabrotica virgifera virgifera virus 2       -----------------------------------NLELKGYNSSSSQYGLFGTPKP--R 3306
OQ540582 Apis mellifera solinvivirus-1                -----------------------------------SLILKGYSSASAQSGLGYAE----- 3356
MN918666 Picornavirales [unknown host]                -----------------------------------NLQLTGYNSASSQFGLIGPRHE--N 3211
MW251313 Icha Creek insect virus                      -----------------------------------LLTRLGYSSPGAQMGNIDSLQH--P 3272
MZ822083 Apis picorna-like virus-4                    -----------------------------------KQSLLGYSGNSAQNGNLEGRSR--G 3077
FJ528584 Solenopsis invicta virus-3                   -----------------------------------QMNALGAGSVSAQNGMYTPS----- 3111
MW314636 Myrmica rubra picorna-like virus             -----------------------------------RMELLGRSSSSALSGNVASS----- 2956
OL569508 Corparats virus-2                            -----------------------------------KMQLLGFSNVSSQQGHNQGTQT--G 3449

DQ286292 Rosy apple aphid virus                       LG-----------HP----------DAPRG--ATLPPRSAS--TQTNSPK---------- 3144
OP265432 Penaeus vannamei solinvivirus                TQLSVLPPAYSEHDPFTSSAKPKE-----GNPSPAKPKEGNHPLGT-------------- 3162
MZ210029 Fushun monoleptalauta Solinvi-like virus 1  VG---------PQQQFVQSGKLDYLAD-SGARAPRKPTVNSQNIGSTKIRVPELDTKDRQ 3742
KX024775 Nylanderia fulva virus 1                     LE---------SGRNFSSSGRLLSGTASSSRGGPLVDLTNNHTTQR----------VTND 3465
KY070327 Diabrotica virgifera virgifera virus 2       GG---------TAV------------------APPPRLITSSGVQTESQQ-----TP--- 3331
OQ540582 Apis mellifera solinvivirus-1                ---------------------------------PNKNFVYSHVYQPNQGP-----QPKEV 3378
MN918666 Picornavirales [unknown host]                QM---------NTRP----------------YQNMNNFVYSHTVTPSVPA-----PTGP- 3240
MW251313 Icha Creek insect virus                      ET---------PAVEK-----EINNSE--N-SIQLPNYF----------R-----PE--- 3297
MZ822083 Apis picorna-like virus-4                    ES---------VQRPP-----ILGVEA--NGMIPRVPLQ----------S-----GTKTE 3106
FJ528584 Solenopsis invicta virus-3                   -------------N-----------------YTPLPSYK----------S-----NTTNY 3126
MW314636 Myrmica rubra picorna-like virus             --------------------------------LPPPSYT----------T-----NSLPS 2969
OL569508 Corparats virus-2                            RN---------PND-----------------PLPDLPYQ----------S-----NLYNY 3468

DQ286292 Rosy apple aphid virus                       ----VYTT-------------------------------------------RPTGFYTGP 3157
OP265432 Penaeus vannamei solinvivirus                FDDFSLSTY---ASESTPGDEVLSMSDPPIYSEQSQAIMKQMGHKEGEGLGKQSQGITS- 3218
MZ210029 Fushun monoleptalauta Solinvi-like virus 1  LGDNMYDDLLHNLSL--NAD-----ETP-----------STLA-------GTQKGIY--- 3774
KX024775 Nylanderia fulva virus 1                     LNDFV-GTT--NNNNGMVPG-----EII---AN------KHFGNL-GSV-HSRHGSISGF 3506
KY070327 Diabrotica virgifera virgifera virus 2       ------------------RS-----NLEPLQEN--------------------------- 3341
OQ540582 Apis mellifera solinvivirus-1                LNDKVYRNIS-GEEVSASDD-----EASPVLSEG-------------------------- 3406
MN918666 Picornavirales [unknown host]                -------------QIGNPDS-----RRSSLMSSD-------------------------- 3256
MW251313 Icha Creek insect virus                      FRKPLYAPFFKSAGI-QNPS-----KINPIPSQGENPVPKNVITTEAEIHTETKPRMSGP 3351
MZ822083 Apis picorna-like virus-4                    LRDAY---------------------------------------------EQQRTKFVKP 3121
FJ528584 Solenopsis invicta virus-3                   YNNSVYHT---DNNITNNPS-----N---------TSLTNNINNFNPELFQQQRERMPTP 3169
MW314636 Myrmica rubra picorna-like virus             K---YSD----NASN--------------------------------------------- 2977
OL569508 Corparats virus-2                            P---YYGA---NNST--------------------------------------------- 3477

DQ286292 Rosy apple aphid virus                       MSSDNDKI---------------------------------------------------- 3165
OP265432 Penaeus vannamei solinvivirus                ------------------------------------PIQPDYSDLA-------------G 3229
MZ210029 Fushun monoleptalauta Solinvi-like virus 1  -LNLHNNLMADGIPGKSDEEKQALVK---------------------------------- 3799
KX024775 Nylanderia fulva virus 1                     SDTINDD--------------------------------PRFVKALE----QQNISKIAN 3530
KY070327 Diabrotica virgifera virgifera virus 2       -------------------------------------PGTETS-----------EASLTP 3353
OQ540582 Apis mellifera solinvivirus-1                ------------------------------DEQPIRYPAKEIKTATLLT--NHAPASLQP 3434
MN918666 Picornavirales [unknown host]                ------------------------------SSDSLP---PEIHDVTHM---TENPAHIPP 3280
MW251313 Icha Creek insect virus                      ESTLNPE------------AKEFTPKINS-------TLNPE--------------AAEFK 3378
MZ822083 Apis picorna-like virus-4                    KDSISNYVKVDGATTDETDVDAIPPFLDRFRSVSTAYPEVADRARQSVV----------P 3171
FJ528584 Solenopsis invicta virus-3                   SEAYDNS-------------KGFVPQPGTSKSIATENINPNYKDEEHIYEPIEQQNHEYA 3216
MW314636 Myrmica rubra picorna-like virus             ---------------------SSV-----SNASTTSYVQPRDIEAN-HYEQINYPEHIYE 3010
OL569508 Corparats virus-2                            ---------------------YKLPFTDSSSSSSQSSIGTRYMTNS-NYS------SIYN 3509
```

Accession	Virus	Alignment	Position
DQ286292	Rosy apple aphid virus	--	3165
OP265432	Penaeus vannamei solinvivirus	GKRYPVLGSDPT-----PTNPSGTGL--KSAKAEFPPPAT--------------------	3262
MZ210029	Fushun monoleptalauta Solinvi-like virus 1	---LSTQDIK-----------RNTDP------------------LLNKKNKQGQTPMDKA	3827
KX024775	Nylanderia fulva virus 1	PWNYDTRGPF-SDFEREIA--TGTRE-----------VKASHLDWAREANRQGRQTLDPK	3576
KY070327	Diabrotica virgifera virgifera virus 2	ASEYTTSPETKAEL----TQRMKSGP--------ITKLSQSTPNWTPRALELGEASVNNA	3401
OQ540582	Apis mellifera solinvivirus-1	SKGTVIPVKDLNDFAIDFKNQTGNDL--------FPKASQTFPNAGVTQ----HA-----	3477
MN918666	Picornavirales [unknown host]	PRNVPYPDET----------PTGVDP--------IDP------ALDKTD----HA-----	3307
MW251313	Icha Creek insect virus	PSALMATD-----------------S-ALNLFLG--------------------------	3394
MZ822083	Apis picorna-like virus-4	NLPMPKSD--------------------------EPDDDENI------------------	3187
FJ528584	Solenopsis invicta virus-3	DIDYNAMNISRENK---NSSNFGNVGILDHQYADIDYDAMKIARDQQNSSKFGNVGVLNH	3273
MW314636	Myrmica rubra picorna-like virus	ELNYPTDS------------DKGYDK-IQNSYLRNPAPQKIY----------------NS	3041
OL569508	Corparats virus-2	GQNSSTRDSI-------ISGNSRNSS-TRNSFLDIPSVSNQI----------------ST	3545

Accession	Virus	Alignment	Position
DQ286292	Rosy apple aphid virus	--	3165
OP265432	Penaeus vannamei solinvivirus	------------------------------------LA----------------------	3264
MZ210029	Fushun monoleptalauta Solinvi-like virus 1	SGA---	3830
KX024775	Nylanderia fulva virus 1	LQAHHNQIKNNA-------------GLLKATTPPTTVE----------------------	3601
KY070327	Diabrotica virgifera virgifera virus 2	FNTQPSQDNAMQRTF------GP-AV---AQ-----------------------------	3422
OQ540582	Apis mellifera solinvivirus-1	-----DQ--RNMPTL------LP-NDVLAQQ-----------------------------	3494
MN918666	Picornavirales [unknown host]	-----NFSSTIVHQL------SP-NAYYNQ------------------------------	3325
MW251313	Icha Creek insect virus	--	3394
MZ822083	Apis picorna-like virus-4	--	3187
FJ528584	Solenopsis invicta virus-3	QYAELDFSKNNTRKN----SQILDNSLYSKTQPSSKMIDNSLYGINPNKMVENQNYEPAS	3329
MW314636	Myrmica rubra picorna-like virus	GKTDPNILEKDSRSFANLLDDSPGNSVYDKTNPLV-------------------------	3076
OL569508	Corparats virus-2	QTNQPSITTENI---AVGTQDLP-------PKTRS-------------------------	3570

Accession	Virus	Alignment	Position
DQ286292	Rosy apple aphid virus	--	3165
OP265432	Penaeus vannamei solinvivirus	----------------GGTSGRIAARSATKTALKAVSKAHPALIAADVASQVVKANPTSL	3308
MZ210029	Fushun monoleptalauta Solinvi-like virus 1	-------------------ASHIAPAIGQVN----------DEIMGGM--ADHAYNVLGL	3859
KX024775	Nylanderia fulva virus 1	------------------------------------ETAHPDLVP----ESEA-------	3614
KY070327	Diabrotica virgifera virgifera virus 2	---------------LAGAFKNINDTKEGV------------------------------	3437
OQ540582	Apis mellifera solinvivirus-1	---------------SQQAVNNIAQTK---------------------------------	3506
MN918666	Picornavirales [unknown host]	--	3325
MW251313	Icha Creek insect virus	--	3394
MZ822083	Apis picorna-like virus-4	--	3187
FJ528584	Solenopsis invicta virus-3	MERKNSIYSSNLNSSNNLKFNNIPNFKGPTN--LNISGAKPAGFGSGIIQP-------AI	3380
MW314636	Myrmica rubra picorna-like virus	LG------------------DNKPSTSGKFNQLNSAMAAHPAGWGAVAGQLEKGFNTVA-	3117
OL569508	Corparats virus-2	IE------------------DKMADAKGISNQFSNAIAAHPQGHGAVMAELKSKQMHGSL	3612

Accession	Virus	Alignment	Position
DQ286292	Rosy apple aphid virus	----------------------------------	3165
OP265432	Penaeus vannamei solinvivirus	GHLA----PV--QPMQPTVSLDGR----------	3326
MZ210029	Fushun monoleptalauta Solinvi-like virus 1	SRYENDNSPTMSASPHPNIDFENTYSPADIHNYL	3893
KX024775	Nylanderia fulva virus 1	----------------------------------	3614
KY070327	Diabrotica virgifera virgifera virus 2	----------------------------------	3437
OQ540582	Apis mellifera solinvivirus-1	----------------------------------	3506
MN918666	Picornavirales [unknown host]	----------------------------------	3325
MW251313	Icha Creek insect virus	----------------------------------	3394
MZ822083	Apis picorna-like virus-4	----------------------------------	3187
FJ528584	Solenopsis invicta virus-3	NKYTDFSKPN------------------------	3390
MW314636	Myrmica rubra picorna-like virus	----------------------------------	3117
OL569508	Corparats virus-2	NQEQSSDLPPMLNSS-------------------	3627
